# Factors affecting relative abundance of low-mobility fishing resources: spiny lobster in the Galapagos Marine Reserve

**DOI:** 10.7717/peerj.7278

**Published:** 2019-07-08

**Authors:** Juan Carlos Murillo-Posada, Silvia Salas, Iván Velázquez-Abunader

**Affiliations:** 1 Departamento de Recursos del Mar, Centro de Investigación y de Estudios Avanzados del Instituto Politécnico Nacional, Unidad Mérida, Mérida, Yucatán, México; 2 Pontificia Universidad Católica del Ecuador, Sede Manabí, Ecuador; 3 Universidad Marista, Mérida, Yucatán, Mexico

**Keywords:** Lobster fishery, Relative abundance, Galapagos Marine Reserve, Generalized additive models, Fisher’s behavior, Catch per Unit Effort

## Abstract

Management of low-mobility or benthic fisheries is a difficult task because variation in the spatial distribution and population dynamics of the resources make the monitoring and assessment of these fisheries challenging. We assumed that environmental, spatial, and temporal factors can contribute to the variability of the relative abundance of such species; we used Generalized Additive Models for Location Scale and Shape (GAMLSS) to test this hypothesis using as a case study the lobster fishery (targeting two species) in the Galapagos Marine Reserve, Ecuador. We gathered data on each of the two species of lobster on a monthly basis over seven years, including: (a) onboard observers’ records of catch data, fishing effort, and ground location by trip, and (b) data from interviews undertaken with fishers at their arrival to port, recording the same type of information as obtained from onboard observers. We use this information to analyze the effect of the measured variables and to standardize the Catch per Unit Effort (CPUE) in each case, using the GAMLSS. For both species, the temperature, region, fishing schedule, month, distance, and the monitoring system were significant variables of the selected models associated with the variability of the catch rate. For *Panulirus penicillatus*, CPUE was higher at night than during the day, and for *Panulirus gracilis* it was higher during the day. Increased temperature resulted in a decrease of CPUE values. It was evident that temporal, spatial scales and monitoring system can influence the variability of this indicator. We contend that the identification of drivers of change of relative abundance in low-mobility species can help to support the development of monitoring and assessment programs for this type of fisheries.

## Introduction

Assessment and management of benthic fisheries resources pose challenges associated with the sampling cost or the lack of knowledge about resource distribution, among other factors (Morsan, 2007). Under these conditions, understanding the variables that could influence spatial and temporal changes in relative abundance of the resources is required. Standardized values of Catch per Unit Effort (CPUE) have been used as an indicator of relative abundance and to understand the dynamics of the resources and the fleets that target them (Maunder & Punt, 2004; Szuwalski et al., 2016; Gibson-Reinemer et al., 2017).

The assessment of resources normally relies on using CPUE as an indicator of relative abundance, using either data coming from biological and oceanographic surveys (fisheries-independent data) or from fisheries-dependent records (commercial catches, information from logbooks, and interviews with fishers, among others). The former includes monitoring programs within the areas of distribution of resources, which are generally costlier than the latter (Maunder & Punt, 2004; Damalas, Megalofonou & Apostolopoulou, 2007; Chuenpagdee, 2011; Reyes, Ramirez & Schuhbauer, 2013; Szuwalski et al., 2016; Gibson-Reinemer et al., 2017). The use of fisheries-dependent data has been the most common approach employed for stock assessment and fisheries evaluation, especially in developing countries (Salas et al., 2007; Cardinale et al., 2014; Saldaña et al., 2017).

The CPUE has been calculated from fisheries-dependent data for several species, including some squids (Denis, Lejeune & Robin, 2002; Roa-Ureta, 2012), penaeid shrimp (Bishop, Venables & Wang, 2004), slipper lobster (Duarte, Severino-Rodrígues & Gasalla, 2010), some pelagic species (Walsh & Brodziak, 2015; Lino et al., 2018), and common carp (Gibson-Reinemer et al., 2017), among others. However, in the case of low-mobility species (e.g., lobsters, bivalves and octopuses), the assumption of homogeneous distribution is doubtful, leading to questions of whether stock abundance is proportional to CPUE (Caddy & Seijo, 1998).

Maunder & Punt (2004) contend that the use of raw CPUE data in isolation provides limited information for stock assessment of exploited resources. Several factors have strong implications for the assumption of proportionality between CPUE and stock abundance, including changes in resource distribution, fishing gear (Arreguín-Sánchez, 1996; Gibson-Reinemer et al., 2017), boat characteristics, time of the day when fishing operations are performed, changes in environmental conditions (Maunder & Punt, 2004), as well as those associated with fisher’s behavior patterns (Salas, Sumaila & Pitcher, 2004; Naranjo-Madrigal, Van Putten & Norman-López, 2015). Identification of some of these factors can provide insights on how to improve monitoring and assessment programs where limited capacity (budget or personal) exists (Maunder & Punt, 2004; Salas & Gaertner, 2004; Saldaña et al., 2017). One way to deal with variability of catch rates has been through the standardization of the CPUE, using Generalized Linear Models (GLM) and Generalized Additive Models (GAM) (Denis, Lejeune & Robin, 2002; Maunder & Starr, 2003; Maunder & Punt, 2004; Yu, Jiao & Carstensen, 2013; Campbell, 2015; Walsh & Brodziak, 2015; Montero et al., 2016).

In this study we aimed to disentangle potential drivers that can modify relative abundance of low-mobility species, like lobster, to understand the dynamics of the resources and to standardize the CPUE trends. The research was undertaken in the Galapagos Marine Reserve (GMR), in Ecuador. In this region, the lobster fishery, one of the most important in the area, has been monitored and managed based on raw CPUE data as an indicator of relative abundance. We contend that this knowledge can help to improved monitoring and assessment programs for this type of fisheries.

### Lobster fishery in the Galapagos Marine Reserve

The spiny lobster is caught by a fleet of 344 vessels, of which 188 are fiberglass, 91 are small wooden (<9.5 m of length), and 65 are larger wooden mother boats (<18 m of length). The first and the second type of boats undertake trips that last two days at sea, and fishers can have two or three trips per week. Mother boats are used when fishers need to move to fishing grounds, farther from their home port; these trips could last up to two weeks. All of them take part in diverse fisheries, including the capture of two species of lobster, *Panulirus penicillatus* and *Panulirus gracilis*. Officially, there are about 1,146 fishers recorded with fishing permits who target these two species around the GMR. Fishers operate from the main fishing ports, located in the three main Islands: Baquerizo Moreno (San Cristobal Island), Villamil (Isabela Island), and Puerto Ayora (Santa Cruz Island). The fishing operations can be undertaken by one or two divers, who fish during the day or at night, using the Hookah system (Hearn et al., 2004; Murillo-Posada, Díaz & Bolaños, 2013; Szuwalski et al., 2016). As in some other regions where lobster is captured (FAO, 2006; Moreno, Peñaherrera & Hearn, 2007), only the tail is sold, and the rest of the body is discarded at sea.

Prior to 2017, lobster fishing season (for both species) lasted from September to December. In 2017, the fishing season was modified, with the opening of the season in July, for the next five years (Comisión Técnica Pesquera, 2016). The management plan implemented in the GMR, including regulations for the lobster fishery, was developed through a participative process involving various fishers and boat-owners (Castrejón, 2011; Castrejón & Charles, 2013; Murillo-Posada, Díaz & Bolaños, 2013). However, the raw CPUE data has been used as a direct indicator of the stock abundance of the two species of lobster to define management schemes, without consideration of variation on the CPUE coming from different sources (e.g., population dynamics and the fishing patterns associated with each species), which could generate ineffective management strategies.

It is important to stress that the short spatial movements, cryptic characteristics, and gregarious habits characteristic of lobster (Atema & Cobb, 1980; Hearn & Murillo, 2008), generally do not comply with the assumption of direct proportionality between resource abundance and raw CPUE values (Hilborn & Walters, 1992; Caddy & Seijo, 1998). In addition, differences in fishers’ experience, fishing power of boats, and fishers’ expectations/preferences (regarding risks and profits) can modify the catch rates (Hearn, 2005; Murillo-Posada, Díaz & Bolaños, 2013; Comisión Técnica Pesquera, 2016).

In this study we aimed to identify the relevant factors that contribute to the variability of the relative abundance of each of the lobster species, considering that spatial, temporal and environmental factors could contribute to changes in the CPUE of the species; changes in fishers’ behavior were also considered as relevant factors associated with the variability of lobster relative abundance. We expect that standardized CPUE could better portray the catch rates trends of each of these resources, and the identification of the factors that contribute to changes in the relative abundance of lobster can help to improve knowledge to support management decisions and to advance in the monitoring systems design applied in the region.

## Methods

### Study area

**Figure 1:**
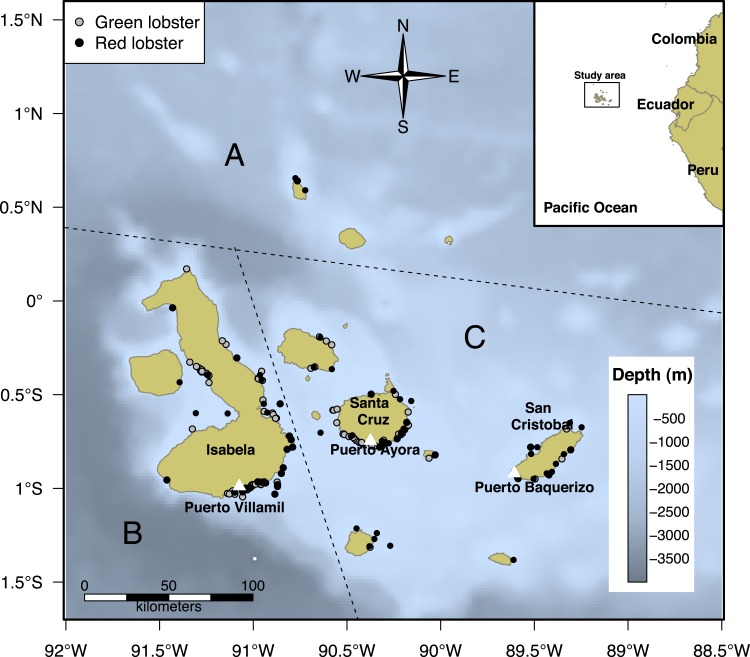
Biogeographic division of the Galapagos Islands by region: North (A), West (B), and Centre-South (C); modified from Edgar et al. (2004). The black dots represent fishing trips in the grounds where *Panulirus penicillatus* (red lobster) was captured by fishers in the area and the gray dots correspond to trips directed to *Panulirus gracilis* (green lobster). Information about distribution of both species represented by black and gray dots was recorded by the onboard observers during the monitoring program in the study period (2002–2008).

The GMR covers an area of about 138,000 km^2^ (http://www.galapagos.gob.ec/reserva-marina/). The lobster fishery has been operating in the area since 1964 (Quiroga & Orbes, 1964). For the spatial analysis of the study, we used the biographic zoning proposed by Edgar et al. (2004), within the reserve area to account for the lobster distribution (Reck, 1983; Murillo-Posada, Díaz & Bolaños, 2013). Edgar et al. (2004) divided the GMR into four zones. We selected three of them (North, West, and the Centre-South zones) to define the monitoring program for the study (Fig. 1). *P. penicillatus* is widely distributed, predominantly in the Centre-South and North zones, while *P. gracilis* is more abundant in the West zone.

In the Galapagos Islands, there are two seasons: the drizzle or cold season, influenced mainly by the Peru or Humboldt Current (June to November) and the wet or warm season (December to May), affected by the Panama Current (Banks, 2002). Our study was undertaken during the fishing season, which includes two months of the cold season (September to October) and two months of the warm season (November to December).

To define the study framework and the field work, we identified the sites where fishers concentrate their fishing effort to capture both species of lobster. While there are 13 large islands in the Galapagos, most fishers are concentrated on three of them: San Cristobal, Santa Cruz, and Isabela (Toral-Granda et al., 2002; Murillo-Posada, Díaz & Bolaños, 2013). Thus, the interviews and onboard observations were conducted in the areas associated with those islands.

### Sources of information

The information associated with the lobster fishery that we used in the present study includes two sets of data source: (a) interviews with fishers at the main landing ports (Villamil, Ayora, and Baquerizo Moreno, using semi-structured interviews); and (b) data gathered by onboard observers, who participated in the fishing journey. The fishers interviewed in the landing ports were different from those interviewed on board. For both data sources, we recorded the following information: catch by species (kg), fishing schedule (fishing operation at day or night), the value of the catch (US dollar), fishing effort (hours), and the name of the ports of departure and arrival. We collected the data during the fishing season (September to December) from 2002 to 2008 (Table 1).

We identified the fishing zones and estimated the approximate distance from the different home ports to the fishing sites. We registered a total of 5,086 trips (3,959 from landings and 1,127 from onboard observers) for *P. penicillatus* and 3,193 for *P. gracilis* (2,749 from landings and 437 from onboard observers) during the study period; some trips occasionally included both species. Given the distance and availability of the resource in the North zone, fishers did not reach those areas as frequently as other regions. Hence, the monitoring effort in this zone was more limited (Fig. 1).

To account for environmental conditions, temperature was recorded in two formats: direct observations and satellite data. The first source includes sea surface temperature (SST), obtained by the onboard observers for each fishing trip during the study period and recorded *in situ* using a cask thermometer (T^∘^C ±0.1). Indirect data sources comprise average monthly temperatures from the MODIS aqua sensor, with level 3 satellite imagery, with 4 × 4 km resolution obtained in hdf5 format (https://oceancolor.gsfc.nasa.gov). We used this information in addition to the data from fishers’ interviews at the landing ports.

Under the assumption that local knowledge of fishers could help to identify and validate fishing effort allocation drivers that support the results generated by the models (Daw, Robinson & Graham, 2011; Van Putten et al., 2012; Mangi et al., 2018), we undertook additional interviews in 2017 with fishers on the three main islands where lobster is captured. A total of 85 interviews were undertaken using semi-structured questionnaires. Inquiries regarding the factors affecting catch rates and incentives to select specific species or fishing sites were included in the questionnaire.

**Table 1:** Number of trips recorded in each region during the monitoring program undertaken in the Galapagos Marine Reserve from 2002 to 2008 for both species of lobsters. Records include information from all ports.

	*Panulirus penicillatus*			*Panulirus gracilis*		
Year	Centre-South	North	West	Centre-South	North	West
*Interviews at Landing*						
2002	636	16	506	228		664
2003	701	7	354	262	3	512
2004	68		159	3		237
2005	39	1	5	9		4
2006	18	5	57	10		52
2007	439	2	315	116		437
2008	281	1	21	22		19
Sub-total	2182	32	1417	650	3	1925
*On board observers*						
2002	208	41	108	19		94
2003	71	24	77	14		81
2004	134		65	19		70
2005	234	20	123	47	2	91
Sub-total	647	85	373	99	2	336
Total	**2829**	**117**	**1790**	**749**	**5**	**2261**

### Selection of variables

As suggested by Anderson (2007), we defined *a priori* 15 candidate models including meaningful variables according to the information reported in the literature, to offer theoretical support regarding the selected components (Defeo et al., 2013; Edgar et al., 2004; Hearn et al., 2004; Moya, Jacome & Yoo, 2017). The implicit assumption we made was that fishers could modify the fishing operations in terms of time and space, given the abundance of the resources, access to those resources, fishers’ experience, and preferences of the fishers for some fishing areas, as well as environmental conditions. We further assumed that these decisions could influence their catch rates. As shown in Table 2, we incorporated in the analysis several components (integrating one or several variables) that could have a potential effect on resource abundance and/or on the diving operations of fishers, that could explain variation in relative abundance of lobster. These components were: spatial (regional and by home port); temporal (fishing schedule, months, years); and environmental, including changes in SST. We also assumed that the monitoring system (using the information from the interviews at the landing sites and onboard observers), could be a source of variation, generating differences in the information recorded (quantity and quality) and thus it could influence the estimates. We defined the CPUE (kg-tail diver^−1^h^−1^) according to the time (h) fishers spent diving during each trip.

**Table 2:** Variables selected to run the standardization analysis of the Catch per Unit Effort for the lobster species targeted in the Galapagos Marine Reserve. The components included in the selection of the final model were: environmental, temporal, spatial, and monitoring system. It was assumed that these components were associated with the behavior of the resource (lobsters), the behavior of the fishers, and the type of data collected.

**Component**	**Variable/indicators**	**Domain**	**Potential effect**
Environmental component	Sea Surface Temperature (SST)	°C	Resources: molting processes, recruitment patterns, and resource distribution could be affected by changes in temperature (William, Lellis & Russell, 1990; Young, Peck & Matheson, 2006; Bermudes & Ritar, 2008)
Temporal component	Month (M) Fishing schedule (F) Year (Y)	Sep–Dec Day or night 2002–2008	Fishing operations: seasonality (annual, monthly) could influence forms of operations. The fishing schedule can be adapted to resources behavior, and fishing patterns can vary given access to resources (Bucaram et al., 2013; Espinoza, Masaquiza & Moreno, 2015; Szuwalski et al., 2016)
Spatial component	Fishing zone/Region (R) Distance (D) Fishers’ homeport (H)	North, Centre-South, West Distance from the homeport to the fishing ground (Km) Baquerizo Moreno, Ayora, Villamil	Fishers’ behavior: fishing operations and homeport distance, accounting for risk, travel costs, resource abundance and fishermen experience (Daw, 2008; Castrejón & Charles, 2013; Naranjo-Madrigal, Van Putten & Norman-López, 2015)
Monitoring system	Data Source (S)	On board observer/ At landing site data collection	Data collection system: that can vary in terms of quality and quantity of data (Espinoza, Masaquiza & Moreno, 2015; Mangi et al., 2018)

We performed multicollinearity tests to ensure that the selected variables of the set of models were not correlated using the Generalized Variance-Inflated Factor (GVIF) method and Principal Component Analysis (PCA) (Fox, 2008; Zuur, Ieno & Elphick, 2010). We present the results of these tests in supplemental information (Table S1; Figs. S1 and S2). Given the observed multicollinearity effect among some of the initially selected variables, we had to choose those that could be used for the candidate models in further analysis, according to the research question and the hypotheses proposed. Based on the test results and the hypotheses stated initially, we discarded year from the variables included in the analysis to build the models, due to its strong correlation with the sea surface temperature. We gave weight to the sea temperature as a relevant factor influencing the abundance and distribution of the resources. According to several authors, temperature affects the physiology and behavior of lobsters like other arthropods (Florey & Hoyle, 1976; Young, Peck & Matheson, 2006; Qadri et al., 2007); these effects could be reflected hence in the distribution and abundance of the lobsters.

To build the candidate models, we added one covariate at a time, using a forward approach to choose the explanatory variables, until completing the 15 fitted models; the order in which we added the covariates was accounting for their explained deviance (expressed in percentage). Among these models, we considered several interactions with potential effect on CPUE variability, such as SST and region, as well as distance to the fishing grounds and the month, if fishers operate more efficiently when climate conditions were more favorable (seasonal effect). Fishers reported in the interviews that during the months of rough winds, when currents are stronger, it is more difficult to navigate and to dive, so they are more inclined to visit closer sites to their home ports for security.

### CPUE standardization

To choose the approach for the selection for the best model that explained the contribution of the explanatory variables that could influence the CPUE of the analyzed species, we compared the Generalized Linear Models (GLM) and the Generalized Additive Models for Location Scale and Shape (GAMLSS) in each case (Hastie & Tibshirani, 1990; Denis, Lejeune & Robin, 2002; Campbell, 2004; Maunder & Punt, 2004; Wood, 2006). The second approached proved to be more appropriate for the data (Table S2), thus we used it for further analyses.

Once we defined the type of model approach to use for each species, we tested different distributions; we assumed that the response variable (CPUE) could be adjusted to some form of the exponential distribution family (e.g., exponential, gamma, and lognormal; see Hastie & Tibshirani, 1990). The gamma distribution was appropriate for both species (See Table S3 and Table S4). This type of distribution has been widely used to analyze CPUE in empirical models (Campbell, 2015); the logarithmic link function *η* = ln(µ) was used in the analysis.

To select the best of the 15 candidate models for of each species (Burnham & Anderson, 2002), we considered the information theory approach, based on the parsimony principle according to the criterion of Akaike (AIC) and the total explained deviance (Hastie & Tibshirani, 1990; Akaike, 1992; Burnham & Anderson, 2001; Venables & Dichmont, 2004; Anderson, 2007). The best candidate model selected was the one with the lowest AIC value.

According to Burnham & Anderson (2002), when the selected models reach similar or close AIC values, the selection is done over those that fit the criteria of Δ_*AIC*_, _*i*_ ≤ 2 with respect to the best model (lowest AIC), assuming they are statistically viable models. All the models that were statistically viable were evaluated with a test of a likelihood ratio test (LRT), in order to compare the goodness-of-fit between them (Hilborn & Mangel, 1997) to provide additional support for choosing the model with greater parsimony (Anderson, 2007).

Once the most parsimonious model was selected, we built the empirical models for the lobster fishery, to test the hypotheses regarding the contribution of the factors associated with the CPUE variability of the species, using the probability (*P)* of the *t*-student test. The final empirical model helped to make an inference of the standardized CPUE for each of the species. To detect temporal changes in CPUE, we contrasted the standardized CPUE values on a monthly basis with the raw CPUE data for both species, integrating information by islands and regions.

All models were run with *R* software (R Core Team, 2018), *glm* function was used for built GLM models while *gamlss* package was used for GAMLSS models (Rigby & Stasinopoulos, 2005).

## Results

### Selecting the models

Regarding the initial analysis contrasting the GLM and GAMLSS models, lower AIC values were attained with the GAMLSS models (Supplementary material shown in Table S2). Hence, we followed this approach for the subsequent analysis, considering it as the most suitable to obtain the best model and to identify relevant variables that contribute to the variability of the lobster CPUE (Table 3).

**Table 3:** Alternative models tested and selection of the best GAMLSS models for the standardization of CPUE in *Panulirus penicillatus* and *P. gracilis* captured in the Galapagos Marine Reserve.

	DF	AIC	Models	Explained deviance (%)
*P. penicillatus*				
**1 (Final)**	**26**	**10925**	**sc(D,3) + SST + R + F + S + M**	**25.0**
2	30	10930	sc(D,3) + SST + R + F + S + M + SST*R	25.1
3	32	10931	sc(D,3) + SST + R + F + S + M + M *D	25.1
4	20	10987	sc(D,3) + SST + R + F + S	
5	24	11004	sc(D,3)+ SST + R + F + M	
6	18	11054	sc(D,3) + SST + R + F	
7	24	11117	sc(D,3) + SST + R + S + M	
8	18	11190	sc(D,3)+ SST + R + S	
9	16	11229	sc(D,3) + SST + R	
10	8	11371	SST + R	
11	14	11561	sc(D,3) + R	
12	12	11618	sc(D,3) + SST	
13	6	11695	R	
14	10	11857	sc(D,3)	
15	4	11917	SST	
*P. gracilis*				
1	30	4414	sc(D,3) + SST + R + F + S + M + SST*R	45.4
**2 (FINAL)**	**26**	**4416**	**sc(D,3) + SST + R + F + S + M**	**45.2**
3	32	4420	sc(D,3) + SST + R + F + S + M + M *D	45.4
4	24	4439	sc(D,3) + SST + R + F + M	
5	24	4445	sc(D,3) + SST + R + S + M	
6	20	4644	sc(D,3) + SST + R + F + S	
7	18	4667	sc(D,3) + SST + R	
8	18	4671	sc(D,3) + SST + R + S	
9	16	4707	sc(D,3) + SST + R + S + M	
10	14	4882	sc(D,3) + R	
11	8	4896	SST + R	
12	6	5049	R	
13	12	5448	sc(D,3) + SST	
14	10	5632	sc(D,3)	
15	4	5672	SST	

**Notes.**

DF degrees of freedom sc spline cubic function to fit the model for continuous variables

The Akaikes’ Information Criterion (AIC) for the set of candidate models (1–15) was used as evidence of fit. The best models for both species are depicted in bold font. Variables: Distance (D), Sea Surface Temperature (SST), Region (R), Fishing Schedule (F), Data Source (S) and Month (M). The models were run using gamlss package of R software. A Likelihood ratio test was performed for models 1 and 2 in *Panulirus gracilis* for the selection of the best model. The most parsimonious model was chosen within final models.

As referred to earlier, Home port and Year variables showed collinearity effect when associated with Region and SST respectively, generating high GVIF values when we performed the test (Table S3 and Figs. S1 and S2). Hence, we excluded the Year and Home port variables from the set of candidate models to select the best model from the data.

Given the close values of the AIC between the candidate models 1 and 2 for *P. gracilis* (Table 3), we used LRT to choose the final model. Since there were no statistical differences in goodness-of-fit between the models (*P* > 0.05), we selected the model without interactions as the most parsimonious, (AIC_Min_ = 4414, AIC_model2_ = 4416, Δ_model2_ = 2).

The selected models for each of the species are depicted in Table 3 and summarized as follows:

1. *P. penicillatus*: CPUE ∼ cs(distance,3) + SST + region + fishing schedule + data source + month. (AIC = 10925; Explained Deviance = 25.0%)
2. *P. gracilis*: CPUE ∼ cs(distance,3) + SST + region + fishing schedule + data source + month. (AIC = 4416, Explained Deviance = 45.2%)

where *cs* is the cubic spline function and SST is the surface temperature of the sea.

### Variables that affect the CPUE of two species of lobster

**Figure 2:**
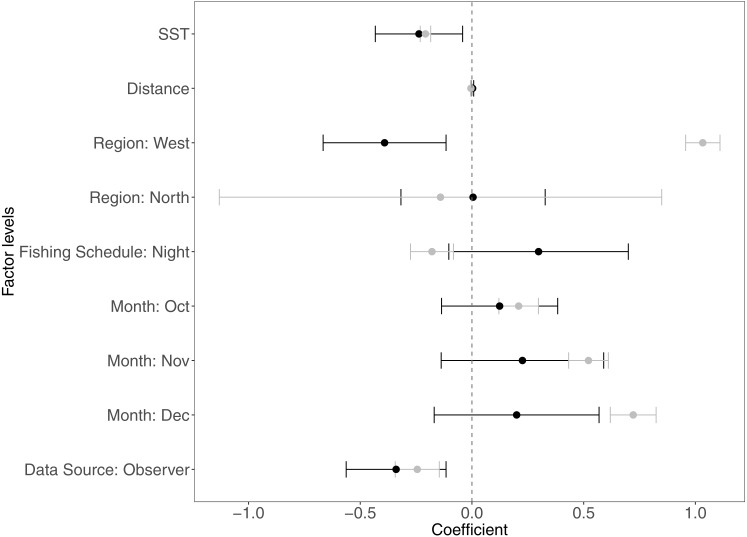
Estimated coefficients of the analyzed variables in the GAM models****for *Panulirus penicillatus* (black dots) and *Panulirus gracilis* (gray dots) and their confidence intervals (CI). The home port coefficients for *Panulirus penicillatus* were excluded because this variable was not significant in the model. SST is sea surface temperature.

The variables that contributed the most to CPUE variability were SST, distance from the fishing site to the home port, the regions, data sources, months, and fishing schedule. In Fig. 2 we present the coefficients estimated by the GAMLSS model for the significant variables for each species. Table 4 depicts the contribution of significant variables to the CPUE variability for each model.

**Figure 3:**
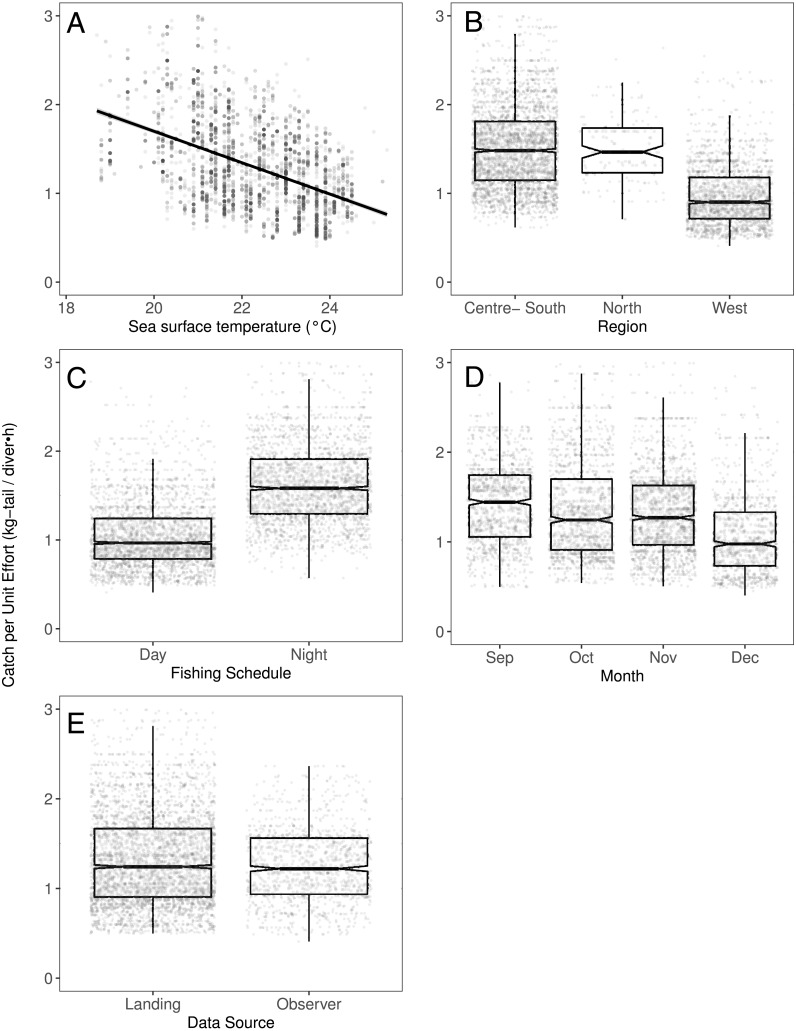
The effect of different variables on Catch per Unit Effort (CPUE, kg-tail/diver h) of *Panulirus penicillatus,* based on the GAMLSS model. (A) Temperature, the solid line represents the CPUE trend standardized by the model, (B) region, (C) fishing schedule, (D) month, and (E) data source. In (B–E) hinges show the interquartile range (IQR: 25% and 75%); the line in the middle of the IQR denotes the median; the upper whisker of the hinge is 1.5 * IQR75% and the lower whisker of the hinge is 1.5 * IQR25%; the notches depict the 95% confidence intervals. Gray dots indicate observed data.

**Figure 4:**
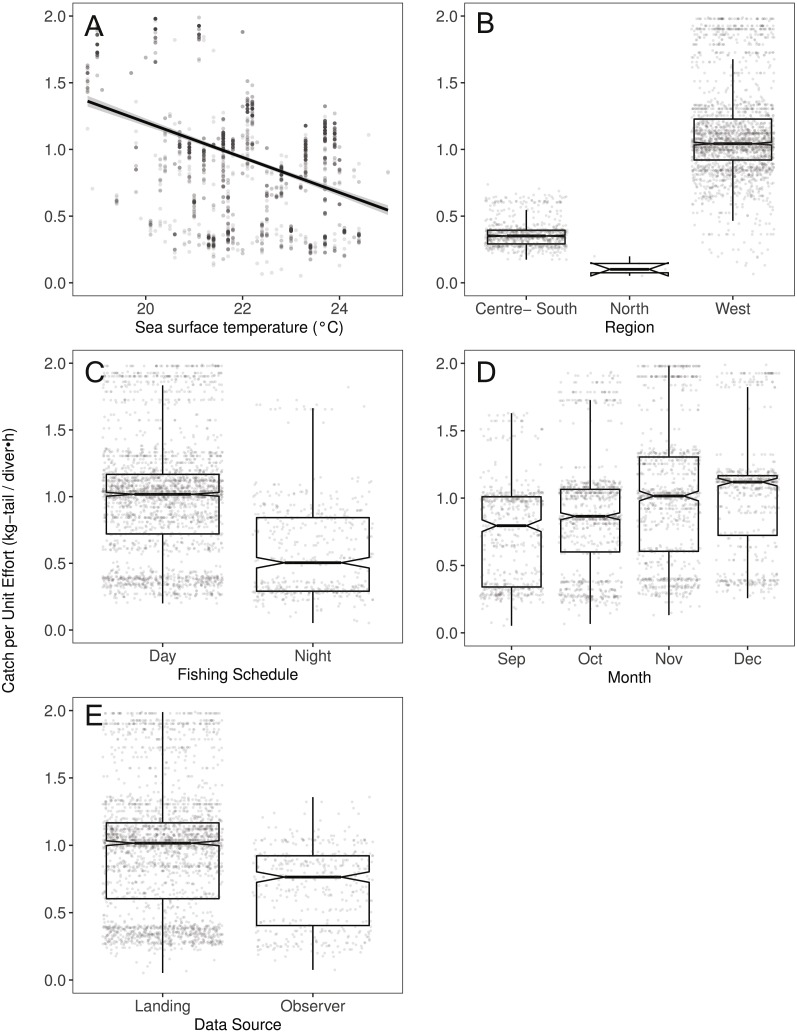
The effect of several variables on Catch per Unit Effort (CPUE, kg-tail/diver h) of *Panulirus gracilis*, based on the GAMLSS model. (A) Temperature, the solid line represents the CPUE trend standardized by the model, (B) region, (C) fishing schedule, (D) month, and (E) data source. In (B–E), hinges show the interquartile range (IQR: 25% and 75%); the line in the middle of the IQR denotes the median; the upper whisker of the hinge is 1.5 * IQR75% and the lower whisker of the hinge is 1.5 * IQR25%; the notches depict the 95% confidence intervals. Gray dots indicate observed data.

**Figure 5:**
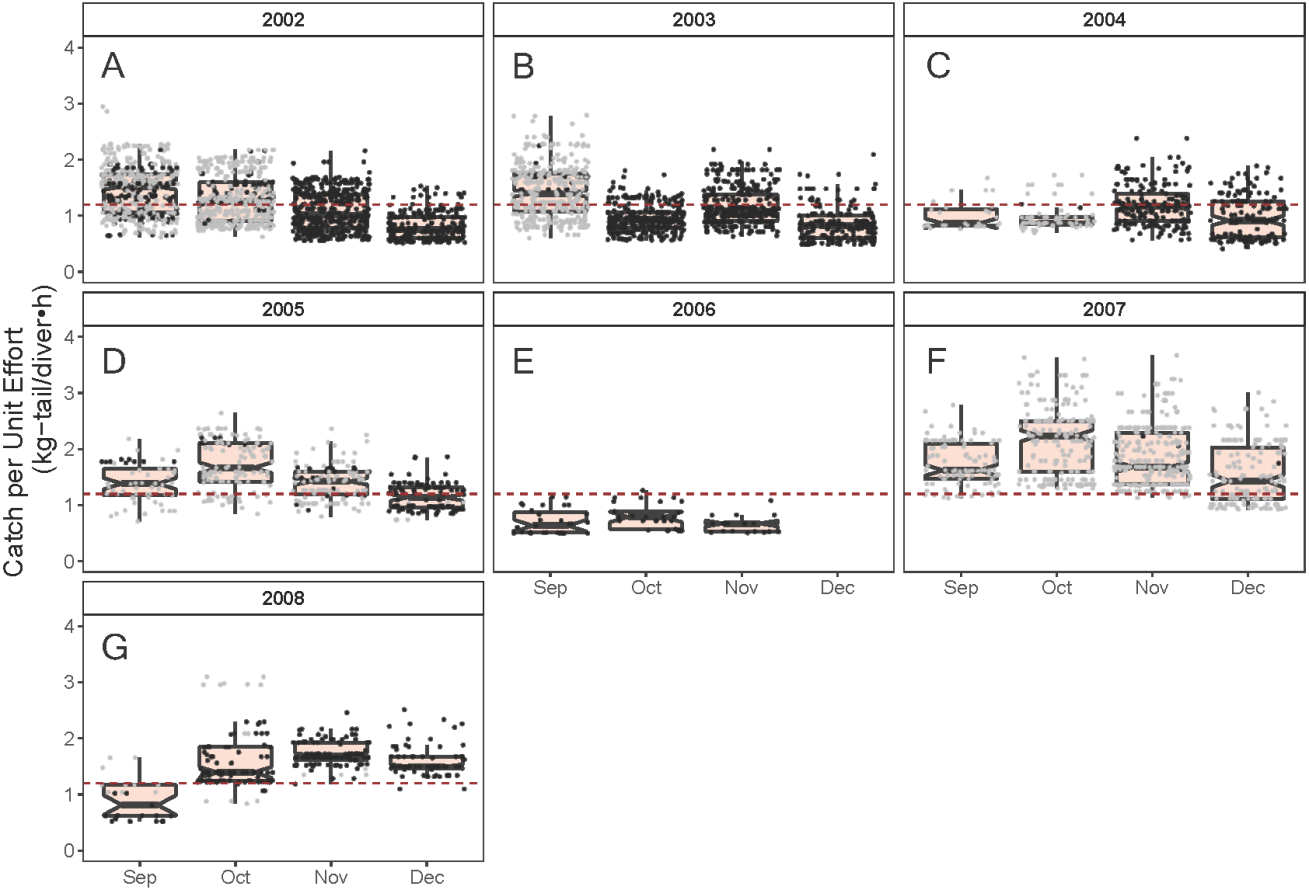
Standardized Catch per Unit Effort (CPUE, kg-tail/diver h) by month of the lobster *Panulirus penicillatus* captured in the Galapagos Marine Reserve for each year of the study period. The black dots represent the mean values of the lobster trips recorded when conditions of the sea surface temperature in the fishing area were above 22 °C. Gray dots represent the trips recorded when sea surface temperature was below 22 °C. The hinges show the interquartile range (IQR: 25% and 75%); the middle line between IQR denotes the median; the upper whisker of the hinge is 1.5 * IQR75% and the lower whisker of the hinge is 1.5 * IQR25%; the notches depict the confidence intervals (95%). The dashed lines represent the general median of the CPUE during the study period (2002–2008).

**Figure 6:**
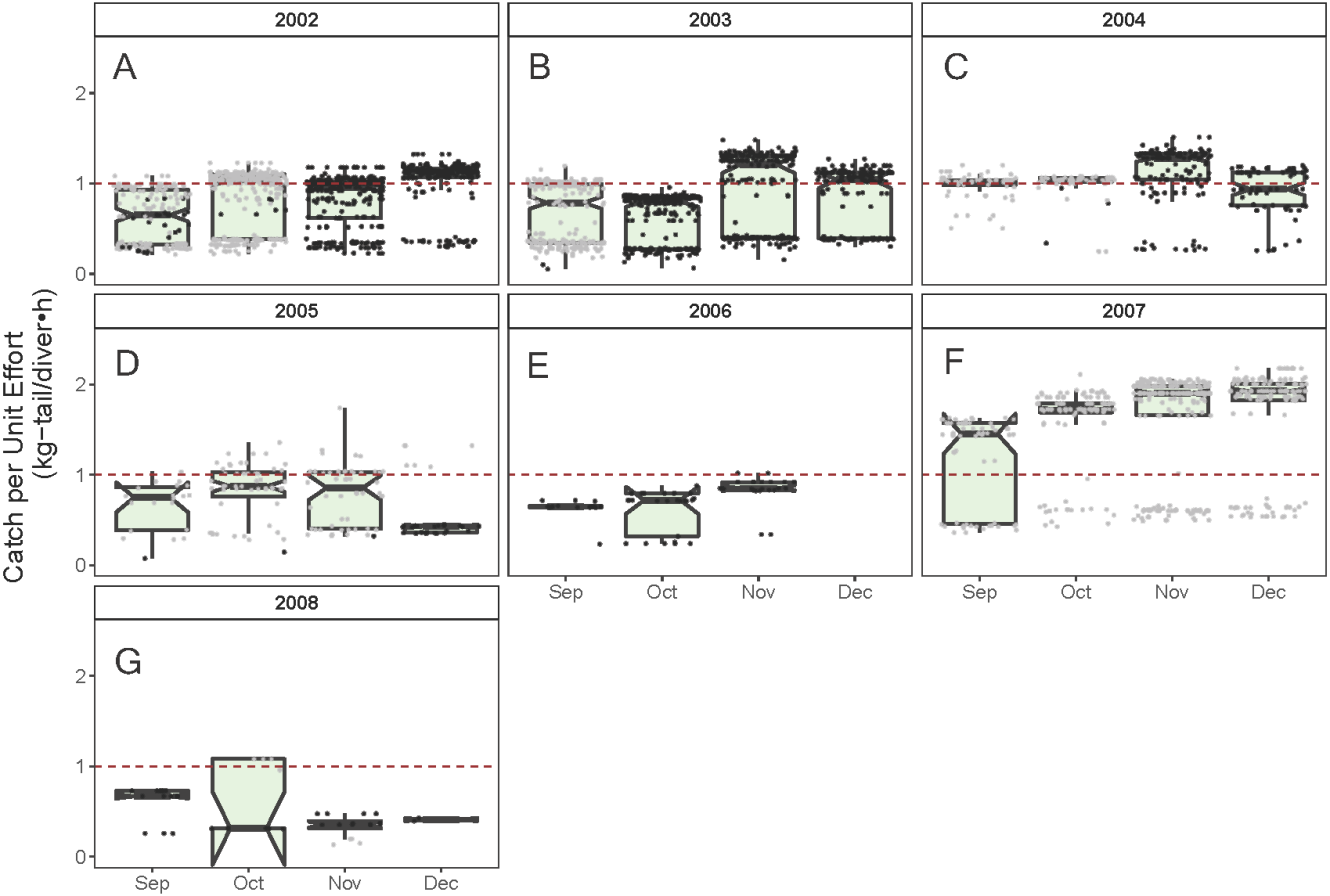
Standardized Catch per Unit Effort (CPUE, kg-tail/diver h) by month of the lobster *Panulirus gracilis* captured in the Galapagos Marine Reserve for each year of the study period. The black dots represent the mean values of the lobster trips recorded when conditions of the sea surface temperature in the fishing area were above 22 °C. Gray dots represent the trips recorded when sea surface temperature was below 22  °C. The hinges show the interquartile range (IQR: 25% and 75%); the middle line between IQR denotes the median; the upper whisker of the hinge is 1.5 * IQR75% and the lower whisker of the hinge is 1.5 * IQR25%; the notches depict the confidence intervals (95%). The dashed red line represents the general median of the CPUE during the study period (2002–2008).

Figures 2–6 illustrate the impacts of the different variables analyzed and explained by each component below.

### Environmental component

We observed that the SST contributed significantly to the lobster CPUE variability for both species in the models (Table 4). The SST recorded in the sites where fishers operated fluctuated between 19 and 25° C. We detected an inverse association between CPUE and SST (see Fig. 3A for *P. penicillatus* and Fig. 4A for the case of *P. gracilis*).

**Table 4:** Results from the Generalized Additive Models for Location Scale and Shape (GAMLSS) for spiny lobsters (*Panulirus penicillatus* and *Panulirus gracilis*) based on information from the period 2002–2008.

	*Panulirus penicillatus*			*Panulirus gracilis*		
	Coefficient	*t* value	*P* (>—t—)	Coefficient	*t* value		*P* (>—t—)	
(Intercept)	4.74	0.23	<0.0001	3.38	0.26	<0.0001
sc(DISTANCE, 3)	0.00	0.00	<0.0001	0.00	0.00	<0.0001
SEA SURFACE TEMPERATURE	−0.22	0.01	<0.0001	−0.21	0.01	<0.0001
REGION: North	0.16	0.08	0.0434	−0.14	0.51	0.7803
REGION: West	−0.33	0.03	<0.0001	1.03	0.04	<0.0001
FISHING_SCHEDULE: Night	0.35	0.03	<0.0001	−0.18	0.05	0.0003
DATA_SOURCE: Observer	−0.28	0.03	<0.0001	−0.24	0.05	<0.0001
MONTH: October	0.19	0.03	<0.0001	0.21	0.05	<0.0001
MONTH: November	0.29		<0.0001	0.52	0.05	<0.0001
MONTH: December	0.28	0.04	<0.0001	0.72	0.05	<0.0001

**Notes.**

*t* value *t*-student test *P* (>|*t*|) statistical significance probability was considered in this study (0.05) sc the spline cubic function used to fit the model for continuous variables

Only coefficients of significant variables that contributed to changes in CPUE were included.

As illustrated in Fig. 5, the monthly CPUE trends of *P. gracilis* showed different patterns among the years when the SST changed, such that when the temperature reached values lower than 22 °C (gray dots), higher CPUE values were obtained, as observed in 2007 (around 2.0 kg-tail diver^−1^ h^−1^). On the other hand, when the SST showed values higher than 22 °C, the CPUE was lower than 1.0 kg-tail diver^−1^ h^−1^ (black dots), as in the years 2006 and 2008. Similar patterns of catch rates were observed for *P. penicillatus,* (Fig. 6).

### Spatial component

Both species were distributed around all the islands in the study site. However, the Centre-South and West regions showed more visits (frequency of trips) targeting *P. penicillatus* (Table 1), the average values of the relative abundance of this species were slightly higher in the North and Centre-South regions (Figs. 2, 3B)*.* In contrast, *P. gracilis* showed higher CPUE values in the West region compared with the other regions (Figs. 2, 4B), consistent with the high number of trips undertaken in this region compared to the rest (Table 1).

**Figure 7:**
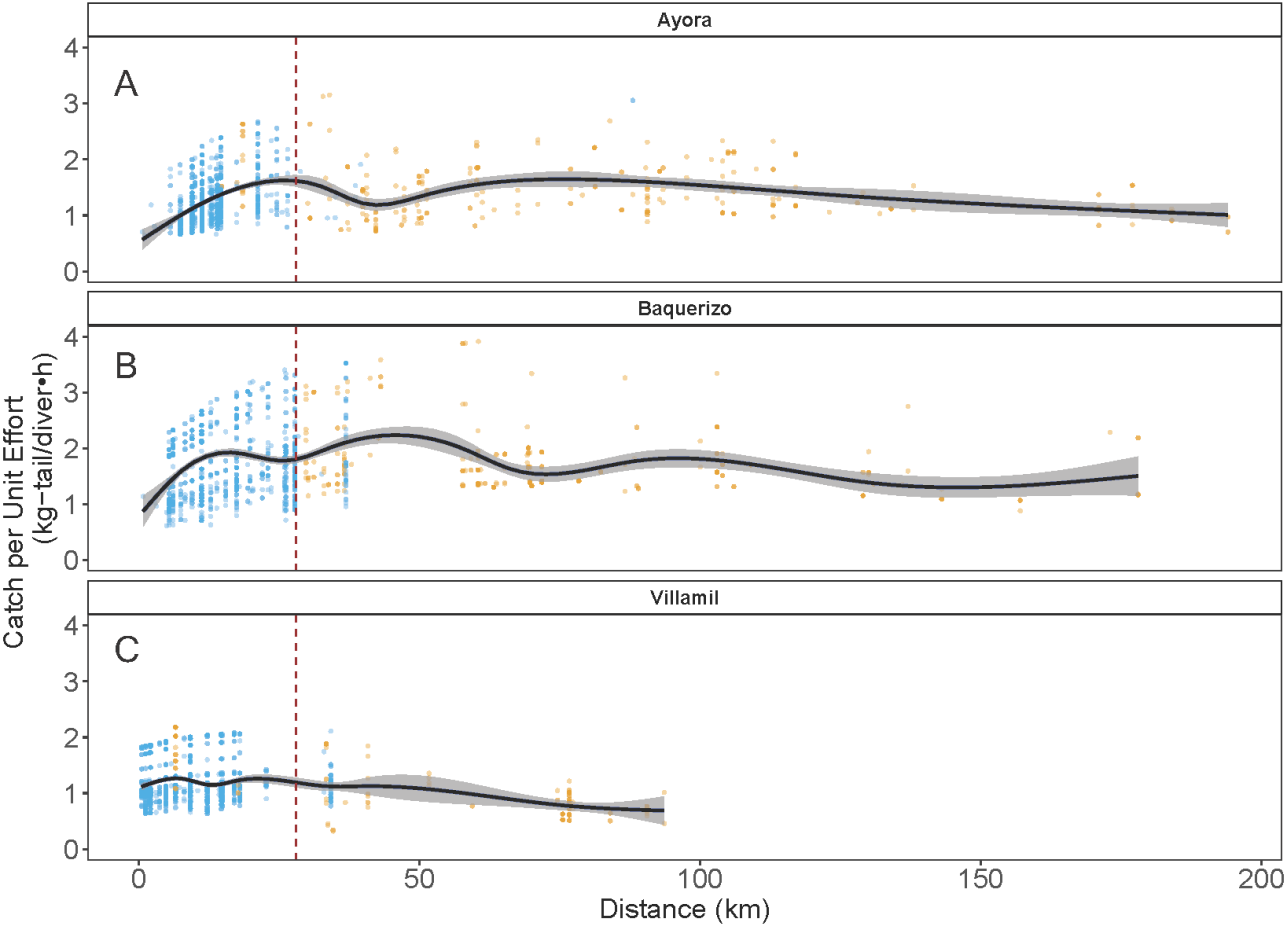
Catch per Unit Effort (CPUE, kg-tail/diver h ) of two species of lobster that occur in the Galapagos Marine Reserve in relation to distance (km) of the fishing grounds from the home port. The blue dots represent the fishing trips within the same island as the fishing port of origin. Gold dots represent trips to other islands. The vertical dashed line indicates the area of highest relative catch rate for each fishing port. Fishers from Ayora Port and Baquerizo Port captured primarily *Panulirus penicillatus* was capture. Fishers from Villamil Port focused on *Panulirus gracilis*.

As indicated earlier, each of the three ports of study is associated with one of the three main islands in Galapagos: Ayora-Santa Cruz, Baquerizo-San Cristobal, and Villamil-Isabela; the other islands are only occasionally visited by fishers. In the three islands, catch rates for both species showed variation in the standardized CPUE values, depending on the distance of the fishing grounds to the home port and the visited islands (Fig. 7). In the case of Ayora and Baquerizo, the CPUE values of *P. penicillatus,* ranged between 1 and 3 kg-tail diver^−1^ h^−1^, within the areas of operation; the lower values were reported in grounds beyond the 150 km. In Villamil, the CPUE values of *P. gracilis* ranged between 1 and 2 kg-tail diver^−1^h^−1^. Initially, fishers tended to visit the closer grounds to their home port, reaching the highest CPUE between the 25 and 28 km from the home port (for Ayora and Baquerizo respectively), and 18 km from the home port for Villamil (see blue dots in Fig. 7). At greater distances, we observed a drop in the CPUE values within the same islands. Some fishers explored new areas beyond this distance with the expectation of increasing their revenues, but the number of trips was low (see gold dots in Fig. 7).

### Temporal component

Regarding the fishing schedule of divers, we observed different patterns in the catch rate of both species; *P. penicillatus* was caught principally during the night (Fig. 3C), and the opposite occurred with *P. gracilis*, for which higher catch rates were observed during the day (Fig. 4C).

For species of low mobility, it has been reported that catch decreases as the season progresses (Cabrera & Defeo, 2001). This applied in the case of *P. penicillatus* as shown in Figs. 2 and 3D, when at the end of the fishing season (December) a decrease in the CPUE was observed. However, the opposite was evident in the case of *P. gracilis*, with an increase in the CPUE as the end of the season approached in November and December (Figs. 2, 4D).

### Monitoring system

There were more data from fishers’ interviews undertaken at the dock (at landing) compared with those obtained from onboard observers (Table 1). The CPUE calculated from landing records were higher than those obtained with information from onboard observers, especially in the case of *P. gracilis* (Figs. 2,  4E). We also observed high dispersion of the CPUE for both species (Figs. 3E, 4E). The differences in the CPUE values according to the two monitoring systems were significant according to the model (*P* < 0.0001; Table 4). To assess the potential effect of lack of information in some of the years when no data were collected with onboard observers (2006–2008), a comparative analysis was undertaken to contrast the standardized CPUE estimated with the two types of monitoring system by year. The result confirmed higher values when data were collected at the landing dock (Fig. S3).

### CPUE standardization

**Figure 8:**
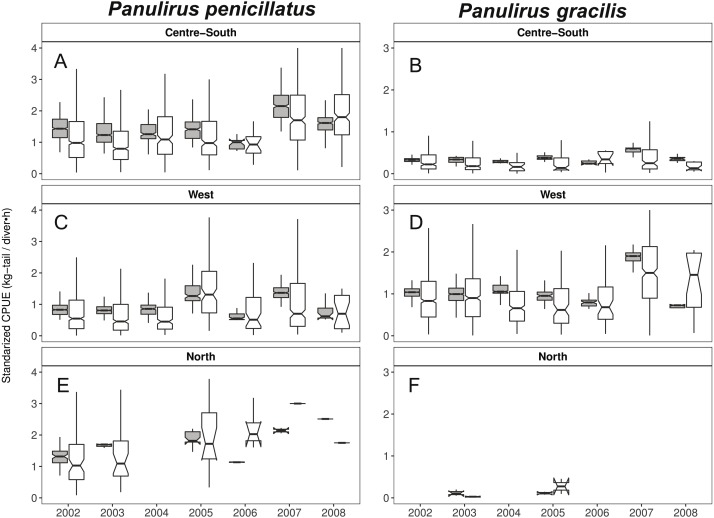
Catch per Unit Effort (CPUE, kg-tail/diver h, white) and Standardized CPUE (gray) in three regions of the Galapagos Marine Reserve from 2002–2008 for *Panulirus pencillatus* and *P. gracilis.* Center-South (A, B); West (C,D); North (E, F). *Panulirus pencillatus* (A, C, E) and *P. gracilis* (B, D, F). The hinges show the interquartile range (IQR: 25% and 75%); the middle line between IQR denotes the median; the upper whisker of the hinge is 1.5 * IQR75% and the lower whisker of the hinge is 1.5 * IQR25%; the notches depict the confidence intervals (95%).

**Figure 9:**
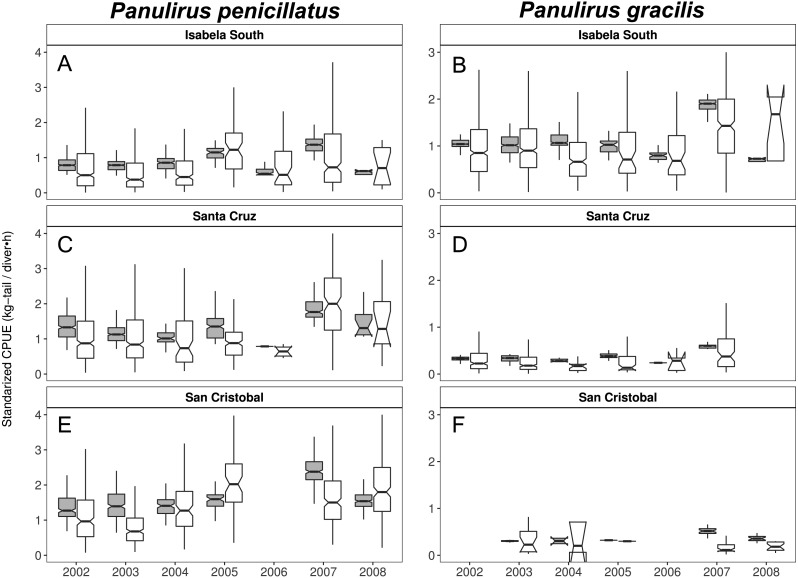
Catch per Unit Effort (CPUE, kg-tail/diver h, white) and Standardized CPUE (gray) by island in the Galapagos Marine Reserve from 2002–2008 for *Panulirus pencillatus* and *P. gracilis.* Isabela South (A, B); Santa Cruz (C, D); San Cristobal (E, F). *Panulirus pencillatus* (A, C, E) and *P. gracilis* (B, D, F).The hinges show the interquartile range (IQR: 25% and 75%); the middle line between IQR denotes the median; the upper whisker of the hinge is 1.5 * IQR75% and the lower whisker of the hinge is 1.5 * IQR25%; the notches depict the confidence intervals (95%).

When looking at the standardized CPUE data for both species at the regional level and by islands, in general, we observed slightly higher values (median and confidence intervals) when compared with the raw CPUE data (Figs. 8, 9). Such differences were more conspicuous in 2007 and 2008 in the Center-South region. This region is one of the most important fishing areas for *P. penicillatus*. In the case of *P. gracilis*, higher values of standardized CPUE were observed in the West region during 2007 (the most important for fishers targeting this species), while the opposite was observed in the year 2008 (Fig. 8). In both cases there was high variability of CPUE, as shown by the interquartile range of raw data. In the North region, the lack of data in some years was noticeable for *P. gracilis,* as depicted in Fig. 8B, because this species is less abundant in that region.

When both species were analyzed at the island scale (associated with the location of the main home ports), relative abundance was higher in San Cristobal and Santa Cruz islands for *P. penicillatus* (Fig. 9A) and in Isabela South for *P. gracilis* (Fig. 9B). In both cases, the differences between the observed and fitted CPUE values were more noticeable between 2007 and 2008.

The total explained deviance for the models of both species was below 50%, indicating that factors other than those considered in this study could contribute to the variability in the CPUE. Future research needs to expand on this subject.

## Discussion

As stated by several authors (Caddy & Seijo, 1998; Caddy & Defeo, 2003; Maunder & Punt, 2004), there is a risk of assuming raw CPUE proportional to resource abundance when assessing commercial or recreational fisheries. This is because a sub-estimation or an over-estimation of abundance can affect the result of the stock assessment and hence can have an impact on the implementation of management strategies. In addition, special care needs to be taken when the fleet targets more than one species, since ignoring the biology, population dynamics, and behavior of the species targeted can generate bias in the assessment that supports management guidance. Such is the case of the lobster fishery in the GMR, where a “resource-based management” orientation from official institutions has coordinated monitoring programs to obtain mainly raw CPUE data to be used as an indicator of lobster abundance (Murillo-Posada, Díaz & Bolaños, 2013; Reyes, Ramirez & Schuhbauer, 2013; Comisión Técnica Pesquera, 2016; Ramírez-González et al., 2018). Pooling information on data of the two species of lobster to support management decisions about the fishery can generate additional bias.

A few studies have addressed the population dynamics of lobsters in the GMP (Hearn & Murillo, 2008; Szuwalski et al., 2016). Standardization of CPUE indicators for the lobster fishery in the region was reported for the first-time by Szuwalski et al. (2016). However, the authors only integrate information concerning *P. penicillatus* in the analysis. In the present study, we run the analysis for each of the species, instead of merging data. The contribution of the variables identified for the models we built for each of the species confirmed the assumptions we made regarding the contribution of environmental, spatial, and temporal factors over the variability of the relative abundance of the species. Here, we expand in each of the components and discuss how the knowledge generated can help to make changes in the monitoring and assessment programs to improve management programs of these types of fisheries.

### The environmental effect

Given the multicollinearity effect observed between the variables year and the *SST* when selecting the relevant variables to build the model for the standardization of the CPUE (Table S1 and Figs. S1 and S2), we choose SST as a relevant factor in this study. We pondered the effect of temperature based on evidence of the importance of this variable on the physiology and biology of the lobster at its larval stage (William, Lellis & Russell, 1990; Matsuda & Yamakawa, 1997; Bermudes & Ritar, 2008). Temperature also plays an important role as a determining factor in survival rate, intermolt period, and growth rates in adult lobsters (William, Lellis & Russell, 1990). These biological processes could influence other aspects such as recruitment patterns in the fishing zones, which in turn could modify the species’ distribution and abundance (Atema & Cobb, 1980; Reck, 1983; Toral-Granda et al., 2002; Daw, 2008; Defeo et al., 2013; Moya, Jacome & Yoo, 2017). All these factors can impact resource availability and catchability, and hence the CPUE values. In the present study, a detailed analysis of the CPUE showed a sensitive response of lobster abundance with small changes in temperature, as illustrated in Fig. 5 and Fig. 6.

Changes in relative abundance of resources can occur among years as reported by several authors (Damalas, Megalofonou & Apostolopoulou, 2007). Although the variable year was not included in the final model, we assert that the interannual variation of relative abundance would be embedded in the standardized CPUE. For instance, it was observed that the highest standardized CPUE values for both species were observed during 2007 (Figs. 8, 9). In this year low temperatures (<22 °C) were recorded throughout the fishing season (Figs. 5, 6).

Other factors can influence interannual variability of CPUE values. For instance, the distribution of lobster has also been associated with the currents in the area, which seem to have an effect on the recruitment patterns as reported by Espinoza, Masaquiza & Moreno (2015). Several authors discuss the relevance of the currents in the Galapagos area and their importance for the resources distributed within the GMR (Reck, 1983; Toral-Granda et al., 2002; Edgar et al., 2004; Murillo-Posada, Díaz & Bolaños, 2013; Moya, Jacome & Yoo, 2017). In the present study, it was not possible to gather enough information about currents, but to improve knowledge, the inclusion of this factor in the expansion of the models could be considered for future research.

### Spatial and temporal components

In fisheries, the selection of an appropriate spatial and temporal scale for assessment and management programs is relevant, especially in the case of benthic species (Cabrera & Defeo, 2001; Morsan, 2007; Castrejón & Charles, 2013; Moya, Jacome & Yoo, 2017). Bias in the estimations of catch rates can have a weak impact on management control. Between 2002 and 2005, a decrease in the CPUE of the lobster close to 30% was reported (Hearn & Murillo-Posada, 2007; Reyes, Ramirez & Schuhbauer, 2013). In the present study, however, the standardized CPUE did not show such patterns. Our results showed an increase of 50% of *P. penicilatus* from 2000 to 2005 in the North and West regions and no change in the Center-South region. The authors cited above did their analysis using raw CPUE, combining information of both species and considering the Galapagos islands as one spatial unit for assessment and management purposes. In the present study, the inter-annual assessment considering the species separately and incorporating the region, distance, and fishing schedule as temporal scales in the standardization process has shown the differences between areas and species. It is important to stress how the knowledge about the dynamics of the fisher’s operations and the changes that occur at different spatial and temporal scales can help to improve our understanding of the factors that could contribute to the variability in the relative abundance of lobsters in the study area.

The indicators generated through the standardization process in this research can be used as an input for stock assessment models to predict trends in biomass of each of the species and to define reference points by species. Spatial planning could also help to improve the implementation of management strategies in the area.

It has been observed in the case of fisheries based on sedentary species, that generally as the fishing season progresses, it is common to observe a decline in relative abundance of the resources, and the restocking of the area is slow in the short-term, given the accumulative effect of the fishing effort and the limited mobility of the animals (Hilborn & Walters, 1992; Caddy & Seijo, 1998). These patterns have been observed in the case of spiny lobster in Mexico as well as other benthic organisms (Caddy & Seijo, 1998; Cabrera & Defeo, 2001). In this study, we observed such behavior in the case of *P. penicillatus*, but not *P. gracilis,* where an increase of the CPUE was evident by the end of the fishing season (November-December).

It is important to recognize that in the Galapagos, clear regionalized climatic patterns have been observed (Wellington, Strong & Merlen, 2001; Edgar et al., 2004). In this research, we observed that fishers from Villamil, who fish mainly *P. gracilus,* have access to a wider area of operation compared with fishers from other ports; they visit mainly the South and West of Isabela island. In this area, the water is generally colder than other potential fishing grounds (21–22 °C) due to the presence of the Humbolt current and the Equatorial Undercurrent (Houvenaghel, 1978; Wellington, Strong & Merlen, 2001; Edgar et al., 2004), apparently favoring an increase on the relative abundance of *P. gracilus.*

As indicated earlier, in 2017, we interviewed fishers, to understand the differences in the observed patterns enquiring about the environmental conditions that could affect the lobster abundance according to their experience. The interviewed fishers stated that they have observed higher abundance of lobster within the fishing season when SST was low. They also indicated that between September and October the intensity of wind and wave energy have a strong effect in their fishing operations. Despite higher abundance can be evident in such period, fishers preferred to fish in calmer water near to shore. During November and December, when conditions improve, they could operate in a more efficient manner in areas of high abundance of lobster. They also reported that lobsters rise from deeper waters and rock reefs when temperature increases in mid-November. Hence, lobsters are observed in the rocky reefs with greater frequency due to immigration of new recruits in the area.

### Fishers’ behavior

In this study, we observed differences in the catches obtained by fishers during the day or at night, which indicate the behavior of lobster and the adaptive strategies developed by fishers from the different home ports when targeting the two available species. When the lobster fishery started in the GMR, fishing at night was not allowed (Reck, 1983). When this restriction was eliminated, some fishers started to operate at night, especially for *P. penicillatus,* as this species has nocturnal feeding habits (Atema & Cobb, 1980; Reck, 1983; Hearn & Murillo, 2008). As stated by one of the interviewed fishers (M Escarabay-Freire, pers. comm., 2017), some fishers take advantage of the lobster behavior to have better access to *P. penicillatusis.* This species is captured mainly at night, especially by fishers from Ayora and Baquerizo Ports. Fishers from Villamil Port, on the other hand, tend to focus mainly on *P. gracilis*, the species that has higher CPUE values, making 91% of their fishing trips for this species during the daytime.

Rent maximization, risk minimization, and socio-cultural patterns, among others, define fishers’ strategies and consequently their operations (Sampson, 1992; Salas, Sumaila & Pitcher, 2004; Bucaram et al., 2013; Naranjo-Madrigal, Van Putten & Norman-López, 2015; Girardin et al., 2017). According to Sampson (1992), fishers would tend to concentrate in the fishing grounds with high biomass, generally located at their reach, near their home ports; hence it is assumed that a direct correlation exists between resource abundance and the distance of the visited grounds. Sampson (1992) emphasizes, for instance, that the cost of traveling would reduce the incentives for fishers to move far from their home ports. Similarly, Anderson & Seijo (2010) refer to the factors that can influence fishers’ operation patterns when selecting their fishing sites, including the “friction of the distance”. This effect assumes that vessels of the limited fishing range tend to inflict a higher impact on fishing areas closer to their home ports, rather than moving to distant zones, especially if they manage to maximize the profit within those areas.

Particularly in the Galapagos islands, the effect of the “frictions of distance” and the incentives referred to by Sampson (1992) were evident with the concentration of the fishing effort around the 25 km of distance of the fishing grounds from the home ports (in Ayora and Baquerizo as depicted in Fig. 5 (blue dots). A positive trend in CPUE was observed from the closer sites, where fishers reached areas with high relative abundance. We define this response to the friction of distance as an “island effect”, since low autonomy of the boats (days at sea) limits engagement in longer trips, which involve higher travel cost.

During the interviews, most fishers indicated that it was more profitable to fish near their home port, due to the type of boats they use. Those who traveled in mother boats to distant islands (North) share their profits with the vessel owners, so they do not perceive the benefits of going farther, if they cannot compensate for the costs of the trip. Other fishers stated that going away to fish in other islands demanded a greater risk of ”not earning as much as expected,” so they preferred to fish near the home port. Bucaram et al. (2013) reported that Galapagos fishing fleet operates based on the expected economic benefits and that would be associated with the travel costs, which was also confirmed in this study.

Based on the results of this study and literature review (Reck, 1983; Hearn et al., 2004; Castrejón, 2011; Bucaram et al., 2013), we could state that in the case of the lobster fishery in the GMR, fishing effort allocation could be explained by: (a) ability to catch species with different behavior (diurnal or nocturnal); (b) the boat autonomy, which is lower in the case of small boats (two days trips) and that can define their operational capacity; and (c) the uncertainty about the relative abundance of the resource in more distant sites associated with high costs.

The relevance of socio-cultural factors is also key when looking at the fishers’ operations, which in turn can be reflected in catch trends (Van Putten et al., 2012; Naranjo-Madrigal, Van Putten & Norman-López, 2015 and references therein). Fishers’ behavior and knowledge can provide important information to understand fishing dynamics. This field of studies is growing and requires more attention, particularly in the GMR.

### Monitoring system

Data from different sources, while costly to obtain, can generate different results about the resource biomass and its respective trends. For this reason, having information from different data sources could help to validate results and assist in the estimation of appropriate indicators (Daw, Robinson & Graham, 2011; Cardinale et al., 2014; Mangi et al., 2018).

In this study, it was possible to gather an important amount of information for both species by region, considering the type of monitoring systems, distribution of both species, and fishing operations around the islands (Table 1). The CPUE standardized values obtained from the data collected through observers on the boats were lower than those coming from interviews at the landing sites. The variation between the sources of information could be because, when observers were onboard, fishers may not operate as freely and may behave differently, modifying their strategies. In general, observations on board of fishers’ boats is more difficult to obtain, demands high costs, and requires cooperation from fishers. However, this type of data is key, as it can help to validate other information collected in the field and on the dock. Information from onboard observers can provide more detailed data, helping to improve understanding of the dynamics of the fishers.

The monitoring system in the GMR has operated for many years, according to a random design, distributed among all islands and in the two types of monitoring systems (interviews at the landing sites and onboard observers). However, our results show the importance of developing a stratified monitoring program design, considering regions, islands, and species, according to the fishing operations in the area. Thus, whenever possible, information from onboard observers should supplement the one collected at the landing ports, in order to improve the quality of data, and to overcome the limitations associated with each method.

### Management implications

Decision-makers need the best available information to implement feasible strategies for fisheries management (Caddy & Seijo, 1998; Salas et al., 2007; Ernst et al., 2010; Naranjo-Madrigal, Van Putten & Norman-López, 2015). The results of this study indicate the importance of the consideration of the spatial scale (region and home port) and the separation of the species in the evaluation of catch rates for improving the understanding of the factors that affect the variability of the resources. This is especially so in oceanic islands, characterized by regions with different microclimates like the Galapagos. Some efforts have been made to define a monitoring plan for sea cucumber in the GMR to improve assessment and deal with the decline of this fishery by accounting for those differences (Castrejón & Charles, 2013). Less has been done to look at the assessments needed for the management of lobster fisheries, which are based on two species. Current monitoring design operates at the level of macro-zones, which is not detail enough (Moreno, Peñaherrera & Hearn, 2007; Comisión Técnica Pesquera, 2016).

Although, the variables in the empirical models generated in this study do not fully explain the variation of the relative abundance of the lobster species at the GMR, these results show the impact of environmental conditions on changes in resources abundance and provide some insights about other relevant factors that play a role in the behavior of fishers and their fishing operations. We stress the importance of defining appropriated monitoring programs that can facilitate the collection of relevant data, broadening the range of factors that could influence variability in the CPUE of benthic species like lobster.

## Conclusions

For the case study of the lobster fishery in the GMR, the use of annual raw data of CPUE as an indicator of abundance to support management schemes has been common. However, given the nature of sedentary species like lobster, where the assumption of homogeneous distribution does not apply, standardization processes of CPUE are required. In this study, even though the total explained deviance by the models of both species did not surpass 50%, the hypothesis regarding the effect of spatial, temporal, and environmental components on the relative abundance of lobster species was confirmed. These results were also consistent with the responses of fishers during the interviews, regarding relevant factors associated with lobster’s abundance and their fishing operations. It is important to acknowledge that this type of study can also provide some insights regarding the strengths and weaknesses of the monitoring systems in place and hence the adjustments required. In this case study, the need to develop a stratified monitoring program was evident.

On the other hand, the results of the study made clear the need to broaden the knowledge about the population dynamics of each of the species studied and their responses (abundance and distribution) to changes due to different variables, which could affect the recruitment or other processes, and consequently the catch rates. Hence, several questions have arisen as topics for future research, such as: (i) Do cold water flows with shallow thermoclines emerging towards the end of the fishing season of *P. gracillis* favor the immigration of new recruits into the fishing area; (ii) does movement of lobsters from deep and narrow caves to shallower waters occur at a given time, making the crustaceans accessible to fishers; and (iii) how do changes in wind patterns influence fishers’ decisions regarding the time and extension of their fishing trips?

It is also important to emphasize that in this kind of study, and particularly when addressing aspects of fishing operations, some socio-cultural and economic factors could explain the behavior of fishers and the observed catch rates. The knowledge generated can help to move forward in the adaptation of monitoring systems, collecting relevant data at the appropriated spatial and temporal scales, and hence to improve the implementation of feasible management strategies for these types of fisheries.

##  Supplemental Information

10.7717/peerj.7278/supp-1 Table S1 Results from the multicollinearity test using the generalized variance-inflation factor method for the two species of spiny lobster GVIF values greater than 3 were considered important (Zuur, Ieno & Elphick, 2010) and are highlighted in the table in the grey box. The variables with high GVIF values were eliminated sequentially until the GVIF values were less than 3. The *vif* function of the *car* package of R software was used for the GVIF calculations.

10.7717/peerj.7278/supp-2 Table S2 Model selection from the global analysis using the General Lineal Models (GLM) and Generalized Additive Models for Location Scale and Shape (GAMLSS) in the Galapagos Marine Reserve for both species The selected models for both species are in bold. In the selection of the final model only significant and no collinear variables are included in the model.

10.7717/peerj.7278/supp-3 Table S3 Test of goodness fit for CPUE in *Panulirus penicillatus*. A–D is the Anderson-Darling method and AIC is the Akaike method

10.7717/peerj.7278/supp-4 Table S4 Test of goodness fit for CPUE in *Panulirus gracilis*. A–D is the Anderson-Darling method and AIC is the Akaike method

10.7717/peerj.7278/supp-5 Figure S1 Evaluation of collinearity between covariates using the Principal Component Analysis (PCA) biplot method, for the lobster *Panulirus penicillatus*

10.7717/peerj.7278/supp-6 Figure S2 Evaluation of collinearity between covariates using the Principal Component Analysis (PCA) biplot method, for the lobster *Panulirus gracilis*

10.7717/peerj.7278/supp-7 Figure S3 Standardized Catch per Unit Effort (CPUE) of lobster regarding the monitoring system, evaluated for the three main fishing islands in the Galapagos Marine Reserve and for each year of study In Santa Cruz and San Cristóbal the CPUE for *Panulirus penicillatus* were analyzed; while for Isabela, the CPUE was compared between the two monitoring systems for *Panulirus gracilis*

10.7717/peerj.7278/supp-8 Supplemental Information 1 R code

10.7717/peerj.7278/supp-9 Dataset S1 Meta data for *Panulirus gracilis*

10.7717/peerj.7278/supp-10 Dataset S2 Meta data for *Panulirus penicillatus*

10.7717/peerj.7278/supp-11 Dataset S3 Raw data for *Panulirus gracilis*

10.7717/peerj.7278/supp-12 Dataset S4 Raw data for *Panulirus penicillatus*

10.7717/peerj.7278/supp-13 Dataset S5 Catch Per Unit Effort estimated by models for *Panulirus gracilis*

10.7717/peerj.7278/supp-14 Dataset S6 Catch Per Unit Effort estimated by models for *Panulirus penicillatus*

## Additional Information and Declarations

### Data Availability

The following information was supplied regarding data availability: The raw data is available in the Supplemental Files.
