# Supplementary material for: Factors affecting relative abundance of low-mobility fishing resources: spiny lobster in the Galapagos Marine Reserve
Source: PeerJ. 2019 Jul 8;7:e7278. doi: 10.7717/peerj.7278 (PMC6622163; doi:10.7717/peerj.7278)
Supplement: Table S1 — GVIF values greater than 3 were considered important (Zuur, Ieno & Elphick, 2010) and are highlighted in the table in the grey box. The variables with high GVIF values were eliminated sequentially until the GVIF values were less than 3. The vif function of the car package of R software was used for the GVIF calculations. [file peerj-07-7278-s001.docx]

|  | **Panulirus penicillatus** | | | | | | | | |
| --- | --- | --- | --- | --- | --- | --- | --- | --- | --- |
|  | Model 1 (All variables) | | | Model 2 (All - H + Y) | | | Model 3 (All – H - Y) | | |
| **Variables** | GVIF | Df | GVIF^(1/(2*Df)^ | GVIF | Df | GVIF^(1/(2*Df)^ | GVIF | Df | GVIF^(1/(2*Df)^ |
| Distance (D) | 3.0 | 1 | 1.7 | 1.9 | 1 | 1.4 | 2.1 | 1 | 1.5 |
| Sea Surface temperatura (SST) | 5.7 | 1 | 2.4 | 5.4 | 1 | 2.3 | 1.4 | 1 | 1.2 |
| Region (R) | 13.2 | 2 | 1.9 | 2.2 | 2 | 1.2 | 2.2 | 2 | 1.2 |
| Fishing Schedule (FS) | 1.9 | 1 | 1.4 | 1.3 | 1 | 1.1 | 1.2 | 1 | 1.1 |
| Data Source (S) | 2.2 | 1 | 1.5 | 2.1 | 1 | 1.5 | 1.2 | 1 | 1.1 |
| Month (M) | 2.7 | 3 | 1.2 | 2.6 | 3 | 1.2 | 1.3 | 3 | 1.0 |
| Year (Y) | 13.9 | 6 | 1.2 | 10.8 | 6 | 1.2 |  |  |  |
| Homeport (H) | 13.8 | 2 | 1.9 |  |  |  |  |  |  |
|  |  |  |  |  |  |  |  |  |  |
|  | ***Panulirus gracilis*** | | | | | | | |  |
|  | Model 1 (All variables) | | | Model 2 (All - H + Y) | | | Model 3 (All - H- Y) | | |
| **Variables** | GVIF | Df | GVIF^(1/(2*Df)^ | GVIF | Df | GVIF^(1/(2*Df)^ | GVIF | Df | GVIF^(1/(2*Df)^ |
| Distance (D) | 1.4 | 1 | 1.2 | 1.2 | 1 | 1.1 | 1.2 | 1 | 1.1 |
| Sea Surface temperatura (SST) | 17.4 | 1 | 4.2 | 17.2 | 1 | 4.1 | 1.2 | 1 | 1.1 |
| Region (R) | 7.4 | 2 | 1.7 | 1.1 | 2 | 1.0 | 1.1 | 2 | 1.0 |
| Fishing Schedule (FS) | 1.1 | 1 | 1.1 | 1.2 | 1 | 1.1 | 1.1 | 1 | 1.1 |
| Data Source (S) | 1.6 | 1 | 1.3 | 1.6 | 1 | 1.3 | 1.2 | 1 | 1.1 |
| Month (M) | 6.6 | 3 | 1.4 | 6.3 | 3 | 1.4 | 1.3 | 3 | 1.0 |
| Year (Y) | 25.9 | 6 | 1.3 | 24.4 | 6 | 1.3 |  |  |  |
| Homeport (H) | 8.0 | 2 | 1.7 |  |  |  |  |  |  |
