# Supplementary material for: Factors affecting relative abundance of low-mobility fishing resources: spiny lobster in the Galapagos Marine Reserve
Source: PeerJ. 2019 Jul 8;7:e7278. doi: 10.7717/peerj.7278 (PMC6622163; doi:10.7717/peerj.7278)
Supplement: Table S2 — The selected models for both species are in bold. In the selection of the final model only significant and no collinear variables are included in the model. [file peerj-07-7278-s002.docx]

| ***Species*** | **Model** | GLM |  | **GAMLSS** |  |
| --- | --- | --- | --- | --- | --- |
|  |  | Explained Deviance (%) | AIC | Explained Deviance (%) | AIC |
| *Panulirus penicillatus* | CPUE ~ temperature + distance to port + region + data source + month + fishing schedule | 13.6 | 12302 | **25.0** | **10925** |
|  |  |  |  |  |  |
| *Panulirus gracilis* |  | 27.4 | 5031 | **45.2** | **4416** |
