# Supplementary material for: Factors affecting relative abundance of low-mobility fishing resources: spiny lobster in the Galapagos Marine Reserve
Source: PeerJ. 2019 Jul 8;7:e7278. doi: 10.7717/peerj.7278 (PMC6622163; doi:10.7717/peerj.7278)
Supplement: Table S3 [file peerj-07-7278-s003.docx]

| **Distribution type** | | **A-D** | | **A-D P value** | | **χ^2^** | **χ^2^ P value** | **AIC** |
| --- | --- | --- | --- | --- | --- | --- | --- | --- |
| **Gamma** | | 4.47 | | < 0.001 | | 454.9486 | < 0.001 | **27281** |
|  |  | |  | |  |  |  |  |
| Weibull | | 10.7642 | | < 0.001 | | 395.9488 | < 0.001 | 27340 |
| Logaritmic Gaussian | | 13.0915 | | < 0.001 | | 646 | < 0.001 | 28013 |
| Exponential | | 19.8699 | | < 0.001 | | 565.7228 | < 0.001 |  |
| Maximum extreme | | 47.9028 | | < 0.001 | | 1,346.75 | < 0.001 |  |
| Logistic | | 112.2732 | | < 0.001 | | 2,337.31 | < 0.001 |  |
| Gaussian | | 202.4704 | | < 0.001 | | 2,699.45 | < 0.001 |  |
| Minimum extreme | | 668.3055 | | < 0.001 | | 8,284.54 | < 0.001 |  |
| Uniform | | 6,569.35 | | < 0.001 | | 22,316.49 | < 0.001 |  |
