# Supplementary material for: Factors affecting relative abundance of low-mobility fishing resources: spiny lobster in the Galapagos Marine Reserve
Source: PeerJ. 2019 Jul 8;7:e7278. doi: 10.7717/peerj.7278 (PMC6622163; doi:10.7717/peerj.7278)
Supplement: Table S4 [file peerj-07-7278-s004.docx]

| **Distribution Type** | **A-D** | **A-D**  **P value** | **χ^2^** | **χ^2^**  **P value** | **AIC** |
| --- | --- | --- | --- | --- | --- |
| **Gamma** | 5.7115 | < 0.001 | 743.9608 | < 0.001 | **12892** |
| **Exponential** | 20.9554 | < 0.001 | 810.452 | < 0.001 | 12923 |
| **Logarítmic Gaussian** | 25.6392 | < 0.001 | 979.8075 | < 0.001 | 13324 |
| **Weibull** | 29.4055 | < 0.001 | 832.5307 | < 0.001 |  |
| **Maximum extreme** | 46.6017 | < 0.001 | 1,647.64 | < 0.001 |  |
| **Logistic** | 100.7028 | < 0.001 | 2,833.64 | < 0.001 |  |
| **Gaussian** | 211.4069 | < 0.001 | 3,065.61 | < 0.001 |  |
| **Minimum extreme** | 1,111.81 | < 0.001 | 17,963.94 | < 0.001 |  |
| **Uniform** | 9,483.09 | < 0.001 | 45,391.13 | < 0.001 |  |
