# Supplementary figures and images for: Factors affecting relative abundance of low-mobility fishing resources: spiny lobster in the Galapagos Marine Reserve
Source: PeerJ. 2019 Jul 8;7:e7278. doi: 10.7717/peerj.7278 (PMC6622163; doi:10.7717/peerj.7278)

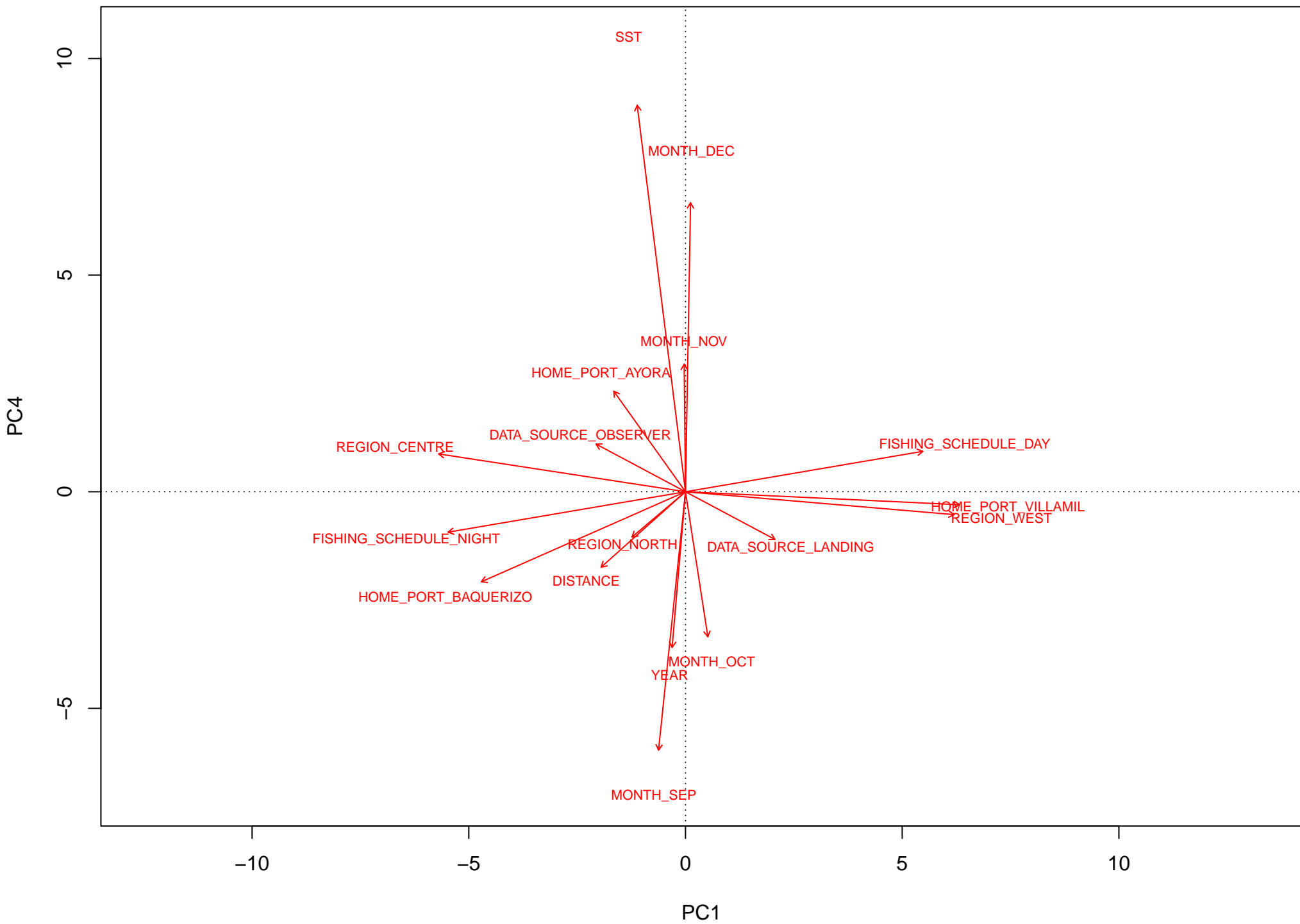

Supplement: Figure S1 [file peerj-07-7278-s005.pdf]

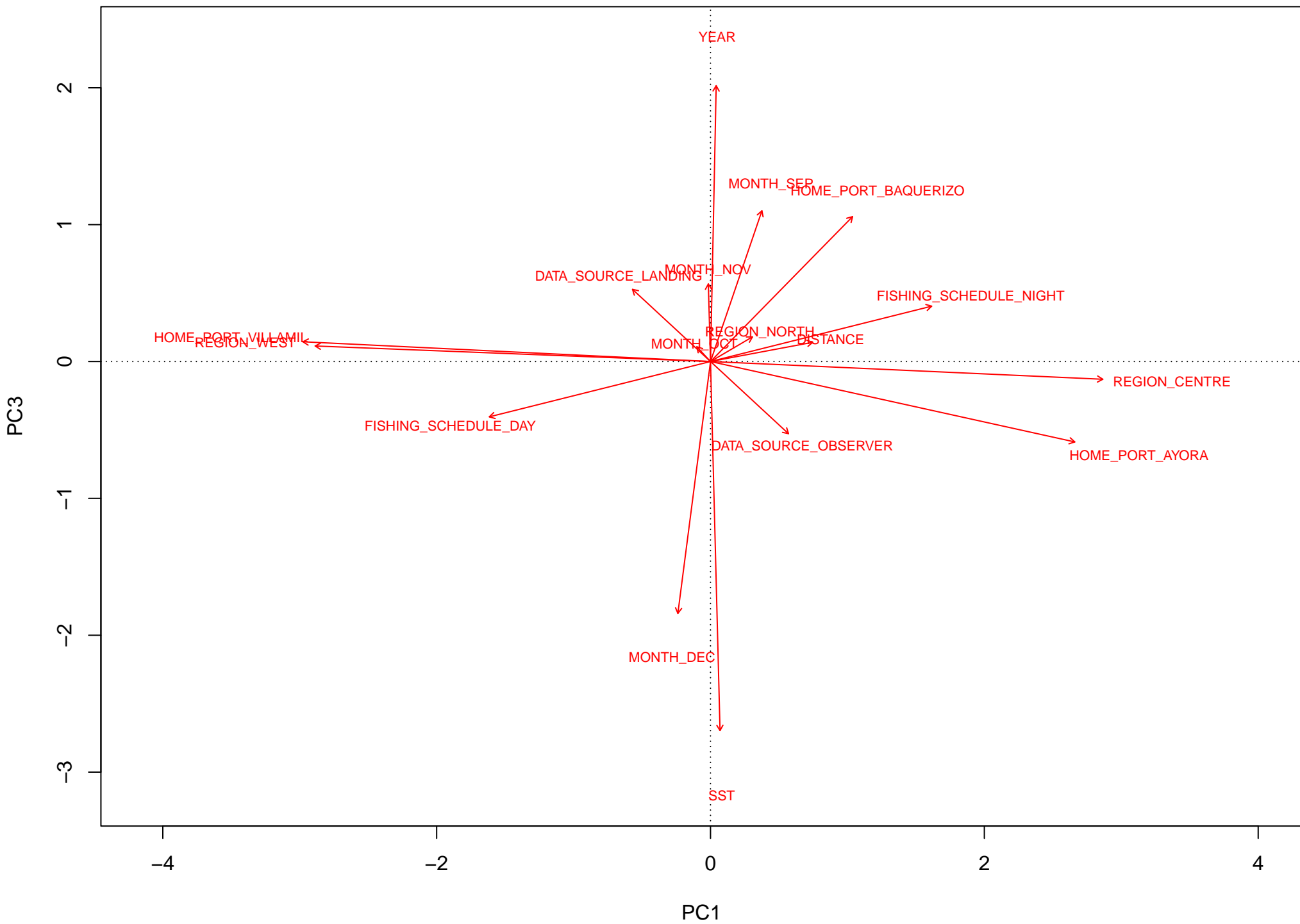

Supplement: Figure S2 [file peerj-07-7278-s006.pdf]
