# Supplementary material for: Factors affecting relative abundance of low-mobility fishing resources: spiny lobster in the Galapagos Marine Reserve
Source: PeerJ. 2019 Jul 8;7:e7278. doi: 10.7717/peerj.7278 (PMC6622163; doi:10.7717/peerj.7278)
Supplement: Figure S3 — In Santa Cruz and San Cristóbal the CPUE for Panulirus penicillatus were analyzed; while for Isabela, the CPUE was compared between the two monitoring systems for Panulirus gracilis [file peerj-07-7278-s007.pdf]

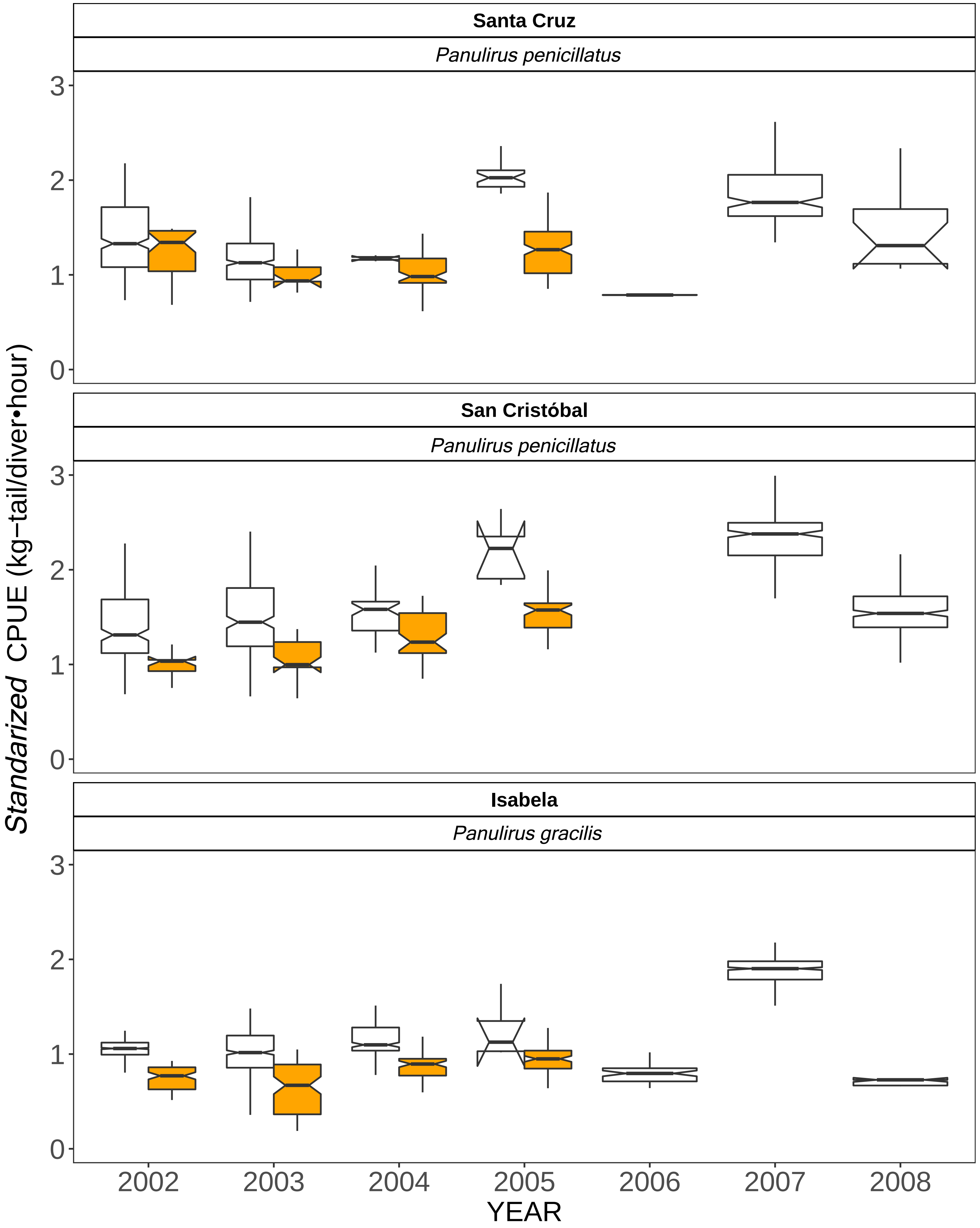

Santa Cruz
Panulirus penicillatus
San Cristóbal
Panulirus penicillatus
Isabela
Panulirus gracilis
Standarized CPUE (kg−tail/diver•hour)
0
1
2
3
2002
2003
2004
2005
2006
2007
2008
YEAR
