# Supplementary material for: Factors affecting relative abundance of low-mobility fishing resources: spiny lobster in the Galapagos Marine Reserve
Source: PeerJ. 2019 Jul 8;7:e7278. doi: 10.7717/peerj.7278 (PMC6622163; doi:10.7717/peerj.7278)
Supplement: Supplemental Information 1 [file peerj-07-7278-s008.docx]

**##### Script for Second Revision of GAMLSS models**

library(mgcv)

library(MASS)

library(gamclass)

library(ggplot2)

library(gamlss)

library(olsrr)

library(AEDForecasting)

library(car)

library(rsq)

library(modEvA)

# Data Base P. penicillatus

setwd("C:/Nuevo E respaldo HP Roja 14032018/Respaldo HP roja 11 julio 2016/Tesis/Analisis de datos/articulo langostas/RESULTADOS/Bases langosta CPUE")

GALR0208<- read.csv("RawData_Red_Lobster.csv", sep=",", dec=".")

head(GALR0208)

GALR0208$MONTH = factor(GALR0208$MONTH, levels=c("Sep", "Oct", "Nov", "Dec"))

GALR0208$YEAR = factor(GALR0208$YEAR)

## Data base P. gracilis

setwd("C:/Nuevo E respaldo HP Roja 14032018/Respaldo HP roja 11 julio 2016/Tesis/Analisis de datos/articulo langostas/Base datos trabajo/Langosta Verde")

GALV0208<- read.csv("RawData_Green_Lobster.csv", sep=",", dec=".")

head(GALV0208)

GALV0208<- read.csv("RawData_Green_Lobster.csv", sep=",", dec=".")

GALV0208$MONTH = factor(GALV0208$MONTH, levels=c("Sep", "Oct", "Nov", "Dec"))

GALV0208$YEAR = factor(GALV0208$YEAR)

head(GALV0208)

#### set candidate models (n=15) fo rP. gracilis

modV1<- gamlss(CPUE ~ cs(DISTANCE, 3), sigma.fo = ~ cs(DISTANCE, 3), family = GA, mu.link="log", data = na.omit(GALV0208))

modV2<- gamlss(CPUE ~ SST, sigma.fo = ~ SST, family = GA, mu.link="log", data = na.omit(GALV0208))

modV3<- gamlss(CPUE ~ REGION, sigma.fo = ~ REGION, family = GA, mu.link="log", data = na.omit(GALV0208))

modV4<- gamlss(CPUE ~ cs(DISTANCE, 3) + SST, sigma.fo = ~ cs(DISTANCE, 3) + SST, family = GA, mu.link="log", data = na.omit(GALV0208))

modV5<- gamlss(CPUE ~ cs(DISTANCE, 3) + REGION, sigma.fo = ~ cs(DISTANCE, 3) + REGION, family = GA, mu.link="log", data = na.omit(GALV0208))

modV6<- gamlss(CPUE ~ SST + REGION, sigma.fo = ~ SST + REGION, family = GA, mu.link="log", data = na.omit(GALV0208))

modV7<- gamlss(CPUE ~ cs(DISTANCE, 3) + SST + REGION, sigma.fo = ~ cs(DISTANCE, 3) + SST+ REGION, family = GA, mu.link="log", data = na.omit(GALV0208))

modV8<- gamlss(CPUE ~ cs(DISTANCE, 3) + SST + REGION + DATA_SOURCE, sigma.fo = ~ cs(DISTANCE, 3) + SST+ REGION + DATA_SOURCE, family = GA, mu.link="log", data = na.omit(GALV0208))

modV9<- gamlss(CPUE ~ cs(DISTANCE, 3) + SST + REGION + FISHING_SCHEDULE, sigma.fo = ~ cs(DISTANCE, 3) + SST+ REGION + FISHING_SCHEDULE, family = GA, mu.link="log", data = na.omit(GALV0208))

modV10<- gamlss(CPUE ~ cs(DISTANCE, 3) + SST + REGION + FISHING_SCHEDULE + DATA_SOURCE, sigma.fo = ~ cs(DISTANCE, 3) + SST+ REGION + FISHING_SCHEDULE + DATA_SOURCE, family = GA, mu.link="log", data = na.omit(GALV0208))

modV11<- gamlss(CPUE ~ cs(DISTANCE, 3) + SST + REGION + FISHING_SCHEDULE + MONTH, sigma.fo = ~ cs(DISTANCE, 3) + SST+ REGION + FISHING_SCHEDULE + MONTH, family = GA, mu.link="log", data = na.omit(GALV0208))

modV12<- gamlss(CPUE ~ cs(DISTANCE, 3) + SST + REGION + DATA_SOURCE + MONTH, sigma.fo = ~ cs(DISTANCE, 3) + SST+ REGION + DATA_SOURCE + MONTH, family = GA, mu.link="log", data = na.omit(GALV0208))

modV13<- gamlss(CPUE ~ cs(DISTANCE, 3) + SST + REGION + FISHING_SCHEDULE + DATA_SOURCE + MONTH, sigma.fo = ~ cs(DISTANCE, 3) + SST+ REGION + FISHING_SCHEDULE + DATA_SOURCE + MONTH, family = GA, mu.link="log", data = na.omit(GALV0208))

modV14<- gamlss(CPUE ~ cs(DISTANCE, 3) + SST + REGION + FISHING_SCHEDULE + DATA_SOURCE + MONTH + SST*REGION, sigma.fo = ~ cs(DISTANCE, 3) + SST+ REGION + FISHING_SCHEDULE + DATA_SOURCE + MONTH + SST*REGION, family = GA, mu.link="log", data = na.omit(GALV0208))

modV15<- gamlss(CPUE ~ cs(DISTANCE, 3) + SST + REGION + FISHING_SCHEDULE + DATA_SOURCE + MONTH + MONTH*DISTANCE, sigma.fo = ~ cs(DISTANCE, 3) + SST + REGION + FISHING_SCHEDULE + DATA_SOURCE + MONTH + MONTH*DISTANCE, family = GA, mu.link="log", data = na.omit(GALV0208))

AIC( modV1, modV2, modV3, modV4, modV5, modV6, modV7, modV8, modV9, modV10, modV11, modV12, modV13, modV14, modV15)

###### likelihood ratio test (LRT)

LR.test(modV13, modV15)

### Final model

modV13<- gamlss(CPUE ~ cs(DISTANCE, 3) + SST + REGION + FISHING_SCHEDULE + DATA_SOURCE + MONTH, sigma.fo = ~ cs(DISTANCE, 3) + SST+ REGION + FISHING_SCHEDULE + DATA_SOURCE + MONTH, family = GA, mu.link="log", data = na.omit(GALV0208))

confint(modV13)

## GVIF for collinearity

glmV17<- glm(CPUE ~ DISTANCE + SST + REGION+ FISHING_SCHEDULE + DATA_SOURCE + MONTH + YEAR + HOME_PORT, family = "Gamma", data=GALV0208)

glmV16<- glm(CPUE ~ DISTANCE + SST + REGION + FISHING_SCHEDULE + DATA_SOURCE + MONTH + YEAR, family = "Gamma", data=GALV0208)

glmV13<- glm(CPUE ~ DISTANCE + SST + REGION + FISHING_SCHEDULE + DATA_SOURCE + MONTH, family = "Gamma", data=GALV0208)

summary(glmV13)

### Explained deviance

rsq.kl(glmV13)

Dsquared(glmV13, adjust=FALSE)

#### GVIF collinearity

vif(glmV17, data=GALV0208)

vif(glmV16, data=GALV0208)

vif(glmV13, data=GALV0208)

### Explained deviance GAMLSS model

Rsq(modV14, type="Cragg Uhler")

Rsq(modV13, type="Cragg Uhler")

Rsq(modV15, type="Cragg Uhler")

#### set candidate models (n=15) for P. penicillatus

modR1<- gamlss(CPUE ~ cs(DISTANCE, 3), sigma.fo = ~ cs(DISTANCE, 3), family = GA, mu.link="log", data = na.omit(GALR0208))

modR2<- gamlss(CPUE ~ SST, sigma.fo = ~ SST, family = GA, mu.link="log", data = na.omit(GALR0208))

modR3<- gamlss(CPUE ~ REGION, sigma.fo = ~ REGION, family = GA, mu.link="log", data = na.omit(GALR0208))

modR4<- gamlss(CPUE ~ cs(DISTANCE, 3) + SST, sigma.fo = ~ cs(DISTANCE, 3) + SST, family = GA, mu.link="log", data = na.omit(GALR0208))

modR5<- gamlss(CPUE ~ cs(DISTANCE, 3) + REGION, sigma.fo = ~ cs(DISTANCE, 3) + REGION, family = GA, mu.link="log", data = na.omit(GALR0208))

modR6<- gamlss(CPUE ~ SST + REGION, sigma.fo = ~ SST + REGION, family = GA, mu.link="log", data = na.omit(GALR0208))

modR7<- gamlss(CPUE ~ cs(DISTANCE, 3) + SST + REGION, sigma.fo = ~ cs(DISTANCE, 3) + SST+ REGION, family = GA, mu.link="log", data = na.omit(GALR0208))

modR8<- gamlss(CPUE ~ cs(DISTANCE, 3) + SST + REGION + DATA_SOURCE, sigma.fo = ~ cs(DISTANCE, 3) + SST+ REGION + DATA_SOURCE, family = GA, mu.link="log", data = na.omit(GALR0208))

modR9<- gamlss(CPUE ~ cs(DISTANCE, 3) + SST + REGION + FISHING_SCHEDULE, sigma.fo = ~ cs(DISTANCE, 3) + SST+ REGION + FISHING_SCHEDULE, family = GA, mu.link="log", data = na.omit(GALR0208))

modR10<- gamlss(CPUE ~ cs(DISTANCE, 3) + SST + REGION + FISHING_SCHEDULE + DATA_SOURCE, sigma.fo = ~ cs(DISTANCE, 3) + SST+ REGION + FISHING_SCHEDULE + DATA_SOURCE, family = GA, mu.link="log", data = na.omit(GALR0208))

modR11<- gamlss(CPUE ~ cs(DISTANCE, 3) + SST + REGION + FISHING_SCHEDULE + MONTH, sigma.fo = ~ cs(DISTANCE, 3) + SST+ REGION + FISHING_SCHEDULE + MONTH, family = GA, mu.link="log", data = na.omit(GALR0208))

modR12<- gamlss(CPUE ~ cs(DISTANCE, 3) + SST + REGION + DATA_SOURCE + MONTH, sigma.fo = ~ cs(DISTANCE, 3) + SST+ REGION + DATA_SOURCE + MONTH, family = GA, mu.link="log", data = na.omit(GALR0208))

modR13<- gamlss(CPUE ~ cs(DISTANCE, 3) + SST + REGION + FISHING_SCHEDULE + DATA_SOURCE + MONTH, sigma.fo = ~ cs(DISTANCE, 3) + SST+ REGION + FISHING_SCHEDULE + DATA_SOURCE + MONTH, family = GA, mu.link="log", data = na.omit(GALR0208))

modR14<- gamlss(CPUE ~ cs(DISTANCE, 3) + SST + REGION + FISHING_SCHEDULE + DATA_SOURCE + MONTH + SST*REGION, sigma.fo = ~ cs(DISTANCE, 3) + SST+ REGION + FISHING_SCHEDULE + DATA_SOURCE + MONTH + SST*REGION, family = GA, mu.link="log", data = na.omit(GALR0208))

modR15<- gamlss(CPUE ~ cs(DISTANCE, 3) + SST + REGION + FISHING_SCHEDULE + DATA_SOURCE + MONTH + MONTH*DISTANCE, sigma.fo = ~ cs(DISTANCE, 3) + SST + REGION + FISHING_SCHEDULE + DATA_SOURCE + MONTH + MONTH*DISTANCE, family = GA, mu.link="log", data = na.omit(GALR0208))

## AIC

AIC(modR1, modR2, modR3, modR4, modR5, modR6, modR7, modR8, modR9, modR10, modR11, modR12, modR13, modR14, modR15)

### likelihood ratio test (LRT)

LR.test(modR13, modR14)

## best model ###

modR13<- gamlss(CPUE ~ cs(DISTANCE, 3) + SST + REGION + FISHING_SCHEDULE + DATA_SOURCE + MONTH, sigma.fo = ~ cs(DISTANCE, 3) + SST + REGION + FISHING_SCHEDULE + DATA_SOURCE + MONTH, family = GA, mu.link="log", data = na.omit(GALR0208))

### confidence intervals

confint(modR13)

#### Collinearity

glmR17<- glm(CPUE ~ DISTANCE + SST + REGION + FISHING_SCHEDULE + DATA_SOURCE + MONTH + YEAR + HOME_PORT, family = "Gamma", data=GALR0208)

glmR16<- glm(CPUE ~ DISTANCE + SST + REGION + FISHING_SCHEDULE + DATA_SOURCE + MONTH + YEAR, family = "Gamma", data=GALR0208)

glmR13<- glm(CPUE ~ DISTANCE + SST + REGION + FISHING_SCHEDULE + DATA_SOURCE + MONTH, family = "Gamma", data=GALR0208)

summary(glmR13)

#### Explained Deviance GLM

rsq.kl(glmR13)

Dsquared(glmR13, adjust=FALSE)

#### GVIF collinearity

vif(glmR17, data=GALR0208)

vif(glmR16, data=GALR0208)

vif(glmR13, data=GALR0208)

#### Summary and coefficients

summary(modR13)

### Explaine deviance for gamlss

Rsq(modR15, type="both")

Rsq(modR14, type="both")

Rsq(modR13, type="both")
